# Natural biased signaling of hydroxycarboxylic acid receptor 3 and G protein-coupled receptor 84

**DOI:** 10.1186/s12964-020-0516-2

**Published:** 2020-02-26

**Authors:** Anna Peters, Philipp Rabe, Petra Krumbholz, Hermann Kalwa, Robert Kraft, Torsten Schöneberg, Claudia Stäubert

**Affiliations:** 1 Rudolf Schönheimer Institute of Biochemistry, Medical Faculty, Leipzig University, Johannisallee 30, 04103 Leipzig, Germany; 2 Rudolf Boehm Institute of Pharmacology and Toxicology, Medical Faculty, Leipzig University, Härtelstraße 16-18, 04107 Leipzig, Germany; 3 Carl Ludwig Institute for Physiology, Medical Faculty, Leipzig University, 04103 Leipzig, Germany

**Keywords:** HCAR, Hydroxycarboxylic acid receptors, GPR109b, GPCR, HCA_3_, GPR84, Dynamin-2

## Abstract

**Background:**

Medium-chain fatty acids and their 3-hydroxy derivatives are metabolites endogenously produced in humans, food-derived or originating from bacteria. They activate G protein-coupled receptors, including GPR84 and HCA_3_, which regulate metabolism and immune functions. Although both receptors are coupled to G_i_ proteins, share at least one agonist and show overlapping tissue expression, GPR84 exerts pro-inflammatory effects whereas HCA_3_ is involved in anti-inflammatory responses. Here, we analyzed signaling kinetics of both HCA_3_ and GPR84, to unravel signal transduction components that may explain their physiological differences.

**Methods:**

To study the signaling kinetics and components involved in signal transduction of both receptors we applied the label-free dynamic mass redistribution technology in combination with classical cAMP, ERK signaling and β-arrestin-2 recruitment assays. For phenotypical analyses, we used spheroid cell culture models.

**Results:**

We present strong evidence for a natural biased signaling of structurally highly similar agonists at HCA_3_ and GPR84. We show that HCA_3_ signaling and trafficking depends on dynamin-2 function. Activation of HCA_3_ by 3-hydroxyoctanoic acid but not 3-hydroxydecanoic acid leads to β-arrestin-2 recruitment, which is relevant for cell-cell adhesion. GPR84 stimulation with 3-hydroxydecanoic acid causes a sustained ERK activation but activation of GPR84 is not followed by β-arrestin-2 recruitment.

**Conclusions:**

In summary, our results highlight that biased agonism is a physiological property of HCA_3_ and GPR84 with relevance for innate immune functions potentially to differentiate between endogenous, non-pathogenic compounds and compounds originating from e.g. pathogenic bacteria.

Video Abstract.

**Graphical abstract:**

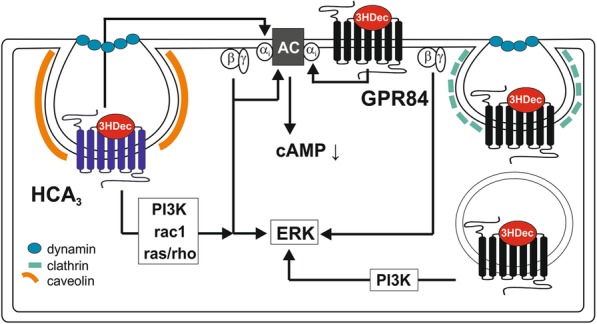

## Background

G protein-coupled receptors (GPCRs) activated by metabolites originating from diet, host- and microbiota metabolism gain more and more attention due to their role as regulators of the host (patho)-physiological state [1–4]. Medium-chain fatty acids (MCFAs), which are saturated fatty acids with 8 to 12 carbons, and their 3-hydroxy derivatives are metabolites acting as agonists at the hydroxycarboxylic acid receptor 3 (HCA_3_) and GPR84 [5–9]. MCFAs originate from medium-chain triacylglycerols present in dairy products and reach plasma levels up to 18 μM, which are further increased under medium-chain triacylglycerol diet [10–17]. 3-hydroxy derivatives of MCFAs are derived from endogenous and exogenous sources. Although basal plasma concentrations of 3-hydroxyoctanoic acid (3HO) and 3-hydroxydecanoic acid (3HDec) rarely exceed 0.4 μM, they are both increased in patients undergoing a ketogenic diet and in patients with diabetic ketoacidosis or defects of mitochondrial β-fatty acid oxidation [5, 11, 18, 19]. 3-hydroxy fatty acids of 10–14 carbon chain lengths are also components of lipopolysaccharides (LPS) of Gram-negative bacteria and have been used as endotoxin markers in clinical and environmental samples [20–23].

HCA_3_ is activated by both, 3HO and 3HDec [5]. 3HDec is also an agonist at GPR84 [7]. Although the role of MCFAs (C10 – C14), specifically decanoic acid (C10), as endogenous agonists at GPR84 is disputed [24], recent work supports their relevance as major endogenous ligands [8, 9]. Both, HCA_3_ and GPR84, are G_i_ protein-coupled receptors and expressed in immune cells, such as neutrophils, macrophages and monocytes [6, 7, 25–27]. GPR84 promotes chemotaxis and pro-inflammatory cytokine release in leukocytes and macrophages [6, 7, 27]. Moreover, chemotaxis of neutrophils and monocytes upon HCA_3_ activation has been reported [25, 26]. However, HCA_3_ is rather suggested to elicit hypo-responsiveness of the immune system through mediation of anti-inflammatory processes [25, 28].

Although HCA_3_ and GPR84 exhibit an obvious overlap in agonist / G-protein specificity and in immune cell expression, their physiological functions appear to be opposed. Therefore, we investigated whether differences in their signaling kinetics and trafficking may explain this apparent contradiction.

We found for both, HCA_3_ and GPR84, that different, yet structurally highly similar agonists have distinct signaling kinetics at the same GPCR, which are not due to differences in their G protein-coupling specificity. We identified differences in Gβγ signaling and in the dependence of receptor internalization on dynamin-2 (dyn-2) and β-arrestin-2 (also named arrestin-3). The distinct agonist- and receptor-dependent recruitment of dyn-2 and β-arrestin-2 triggers activation of different downstream signaling cascades constituting the molecular basis for the observed biased signaling via HCA_3_ and GPR84.

## Methods

### Chemicals

All compounds and inhibitors were purchased from Sigma-Aldrich, Cayman Chemical, Santa Cruz Biotechnology if not stated otherwise.

### Amplification, sequencing and cloning of GPCR and dyn-2 constructs

Genomic DNA was isolated from human cancer cell lines using the Wizard Genomic DNA Purification Kit (Promega). Primer pairs, positioned in the 5′- and 3′-UTR were used to amplify GPR84 (Table S1). Prof. Ralf Jacob kindly provided the wild-type (wt) rat dyn-2 construct [29]. It was used as template to introduce K44A and R399A mutations as well as the YFP-tag (primer pairs in Table S1). Cloning of human, gorilla and orangutan HCA_3_ was previously described [25]. The ProLink Cloning Vector Bundle was purchased from Eurofins DiscoverX and human HCA_3_ and GPR84, respectively were inserted in the pCMV-ProLink Vector (primer pairs indicated in Table S1).

PCR reactions were performed with Q5 High-Fidelity DNA Polymerase following the manufacturer’s instructions. The PCR reaction (50 μl) contained genomic DNA with primers (0.5 μM each), Q5 Reaction buffer (1x), dNTP (200 μM), and 0.02 U/μl Q5 polymerase (NEB, Frankfurt am Main, Germany). The reactions were initiated with denaturation at 98 °C for 30 s, followed by 35 cycles of denaturation at 98 °C for 20 s, annealing at 63 °C for 45 s and elongation at 72 °C for 1 min. A final extension step was performed at 72 °C for 10 min. GPR84 was epitope-tagged with an N-terminal hemagglutinin (HA) epitope and a C-terminal FLAG-tag by a PCR-based overlapping fragments approach and inserted into the mammalian expression vector pcDps. Similarly, rat dyn-2 was C-terminally tagged with YFP and human HCA_3_ C-terminally tagged with mRuby. Identity and correctness of the constructs were confirmed by sequencing (SeqLab). The β-arrestin-2-YFP construct was a gift from Robert Lefkowitz (Addgene plasmid #36917) [30].

### Cell culture and transfection

All cells were maintained at 37 °C in a humidified 5% CO_2_ incubator. The Chinese hamster ovary cell line CHO-K1 (ATCC CCL-61) and the human embryonic kidney cell line HEK293-T (ATCC CRL-3216) were obtained from the American Type Culture Collection. The esophageal squamous cell carcinoma cell line Colo680N (ACC 182) was obtained from the Leibniz Institute DSMZ (German Collection of Microorganisms and Cell Cultures). The CHO-K1 cells stably expressing HCA_3_ (cAMP Hunter CHO-K1 GPR109B Gi Cell Line) were purchased from Eurofins DiscoverX.

CHO-K1 cells were grown in Dulbecco’s Modified Eagle Medium: Nutrient Mixture F-12 (DMEM/F12) and HEK293-T cells in DMEM. Colo680N cells were grown in Roswell Park Memorial Institute (RPMI) 1640 Medium with high glucose. All media were supplemented with 10% fetal bovine serum (FBS), 100 U/ml penicillin and 100 μg/ml streptomycin. CHO-K1 cells stably expressing HCA_3_ were cultured in commercially available AssayComplete Cell Culture Kit 107 Medium supplemented with 800 μg/ml geneticin. The enzyme acceptor- (EA) tagged β-arrestin-2 cell line was generated according to the manufacturer’s protocol (Eurofins DiscoverX). In brief, 1 × 10^6^ HEK293-T cells were seeded in 10 cm culture dishes and infected with PathHunter-β-arrestin-2-EA retroparticles (Eurofins DiscoverX 93–1087) at a concentration of at least 1 × 10^6^ pfu/ml. Cells were subsequently selected for hygromycin resistance (final concentration 500 μg/ml Invivogen ant-hg-5). Individual clones were isolated and tested for the presence of residual viral particles in compliance with BSL-2 safety regulations. Cells considered to be stable and safe were then exploited for subsequent G protein activation assays. HEK293-T cells stably expressing β-arrestin-2-EA were cultured in DMEM, supplemented with 10% FBS and 250 μg/ml Hygromycin B (Thermo Fisher Scientific).

For transient transfection Lipofectamine 2000 (Thermo Fisher Scientific) was used. Cells were split into 25 cm^2^-cell culture flasks (CHO-K1: 0.9 × 10^6^ cells/flask, CHO-K1-HCA_3_: 1.2 × 10^6^ cells/flask, HEK293-T: 1.6 × 10^6^ cells/flask, HEK293-T cells stably expressing β-arrestin-2-EA: 2 × 10^6^ cells/flask, Colo680N: 1.6 × 10^6^ cells/flask). The following day cells were transfected with plasmid (total amount of DNA: CHO-K1: 3 μg, HEK293-T: 2 μg (single construct) or 4 μg (co-transfection), HEK293-T cells stably expressing β-arrestin-2-EA: 0.05 μg, Colo680N: 4 μg).

For cAMP-, ERK-, ELISA-assays, calcium imaging and CQ1 analyses cells were serum-starved 16 h prior to the experiments. In case inhibitors were used, cells were incubated for 30 min at 37 °C prior to the experiment using the following concentrations: 10 μM U0126, 80 μM dynasore, 100 μM barbardin, 3 mM MβCD, 50 μM gallein, 25 μM ZA, 100 μM NSC23766 and 25 μM LY294002.

### 3D spheroid growth analyses

Cells were cultured in ultra-low attachment spheroid microplates (Corning Life Sciences for CHO-K1; Nexcelom for Colo680N) to form spheroids and analyze receptor function in 3D structures. For stimulation experiments cells (CHO-K1 & CHO-K1-HCA_3_: 1 × 10^4^ cells/well, Colo680N: 2 × 10^4^ cells/well) were seeded in their culture medium with or without agonist. For experiments with dyn-2 mutants, cells were seeded 24 h post-transfection with dyn-2 constructs. Images were taken and analyzed every 24 h for up to 72 h using the Celigo Image Cytometer (Nexcelom). At 72 h cells were stained for dead (propidium iodide (PI), 1 μg/ml Thermo Fisher Scientific) and total cells (Hoechst 33342, 1 μg/ml, Sigma-Aldrich) and imaged as well as analyzed using the Celigo Image Cytometer.

### Dynamic mass redistribution assay

To measure label-free receptor activation, a dynamic mass redistribution (DMR) assay (Corning Epic Biosensor Measurements; Corning Life Sciences) with CHO-K1 cells transiently transfected with receptor construct or empty vector was performed. One day after transfection cells were detached using Versene solution (Life Technologies) and transferred into a fibronectin-coated Epic 384-well microplate at a density of 1.2 x 10^4^cells per well and cultured for 24 h to reach confluent monolayers. After 2 h of equilibration in HBSS/HEPES, stimulation with various agonist concentrations was performed and DMR was recorded for 50 min. In DMR measurements, polarized light is passed through the bottom of the biosensor microplate, and a shift in wavelength in pm of reflected light indicates intracellular mass redistribution triggered by receptor activation.

### ALPHAScreen cAMP assay

cAMP content of cell extracts was determined by a non-radioactive assay based on the ALPHAScreen technology according to the manufacturer’s protocol as previously described (Perkin Elmer LAS) [25].

### Alpha *SureFire* Ultra Multiplex pErk 1/2 & total Erk assay

pErk/total Erk content of cell extracts was determined by the Alpha SureFire Ultra Multiplex p-ERK 1/2 + Total ERK assay according to the manufacturer’s protocol (Perkin Elmer LAS). The kit measures both, the phosphorylation (Thr202/Tyr204) and total levels of endogenous ERK 1/2 in cellular lysates. The signal at 615 nm (Eu) corresponds to the phosphorylated ERK level, and the signal at 545 nm (Tb) corresponds to the total ERK levels.

One day after transfection cells were split into 96-well plates (2 × 10^4^ cells/well). Stimulation with agonists was performed 48 h after transfection in HBSS/HEPES for 10 min at 37 °C if not indicated otherwise. When inhibitors were used cells were pre-incubated with inhibitor in HBSS/HEPES at 37 °C for 30 min prior to agonist stimulation. Two-fold concentrated agonist was added to inhibitor-containing wells to prevent wash-out effects. Reactions were stopped by aspiration of media and cells were lysed in 50 μl of supplied lysis buffer. From each well 10 μl of lysate were transferred to a 384-well plate. Acceptor beads and donor beads were added according to the manufacturer’s protocol.

### CQ1 confocal imaging

HEK293-T cells co-transfected with HCA_3_-mRuby or GPR84-mRuby and either YFP-tagged rat dyn-2 variants or YFP-tagged rat β-arrestin-2 were plated in poly-L-lysine treated black Greiner 96-well plates with clear bottom (Greiner No 655090). Forty-eight hours post-transfection, medium was changed to HBSS/HEPES and after 30 min incubation images were acquired using the Yokogawa CQ1 (Cenibra). Subsequently, buffer with or without agonist was added to the cells. Then cells were incubated for another 30 min and images acquired of the same cells. Per condition several images were acquired with a 40x objective and at least 30 cells analyzed.

### ELISA

Cell surface expression of N-terminal HA-tagged receptor constructs was determined using an indirect cellular ELISA as described previously [25].

### Calcium imaging

CHO-K1 and HEK293-T cells were transfected with plasmids encoding for mRuby-tagged HCA_3_ and mRuby-tagged GPR84, respectively. Transfected cells (2 × 10^5^ cells/well) were seeded into 24-well plates on glass cover slips and calcium imaging was carried out 24–48 h post-transfection. CHO-K1 and HEK293-T cells were loaded with 5 μM fura-2 AM (Molecular Probes) in standard solution containing 140 mM NaCl, 10 mM HEPES, 5 mM KCl, 2 mM CaCl_2_, 1 mM MgCl_2_, and 10 mM glucose for 60 and 30 min, respectively. Fura 2-based calcium imaging was performed in single transfected CHO-K1 and HEK293-T cells using a monochromator-based imaging system and the imaging software TILLvisION 4.0 (T.I.L.L. Photonics). Emitted fluorescences (excited at 340 nm and 380 nm) were acquired with a CCD camera (PCO Imaging) at intervals of 2 s and corrected for background fluorescence. Transfected cells were detected by emitted mRuby fluorescence, excited at 550 nm. Agonists of GPR84 and HCA_3_ as well as ATP were dissolved in standard solution and applied to the cells by bath perfusion.

### PathHunter β-arrestin assay (Eurofins DiscoverX)

One day after transfection 5 × 10^3^ cells/well were plated in a poly-L-lysine-treated white 384-well plate with clear bottom (Greiner No 781098). On the day of the assay, media was removed and cells were stimulated with 25 μl of agonist solution in HBSS/HEPES for 90 min at 37 °C and 5% CO_2_. Subsequently, 12.5 μl detection solution was added following the manufacturer’s instructions. After 1 h incubation in the dark at room temperature, luminescence was determined using the EnVision 2105 (Perkin Elmer).

### Data analyses

All data were analyzed and visualized using GraphPad Prism version 7 for Windows (GraphPad Software, www.graphpad.com). A repeated measure one-way ANOVA (Dunnett’s multiple comparisons test) or paired two-tailed t-test was applied to analyze differences in spheroid area and number of cells. Unpaired two-tailed t-tests were applied to analyze differences in cell surface expression. Unpaired two-tailed t-tests were applied to analyze the effect of inhibitors and dyn-2 mutants for cAMP inhibitory signaling. Paired two-tailed t-tests were performed to analyze the effect of inhibitors and dyn-2 mutants on ERK activation. ^#^*P* ≤ 0.1; * *P* ≤ 0.05; ** *P* ≤ 0.01; *** *P* ≤ 0.001.

## Results

### cAMP inhibitory signal of HCA_3_ and GPR84

**Fig. 1:**
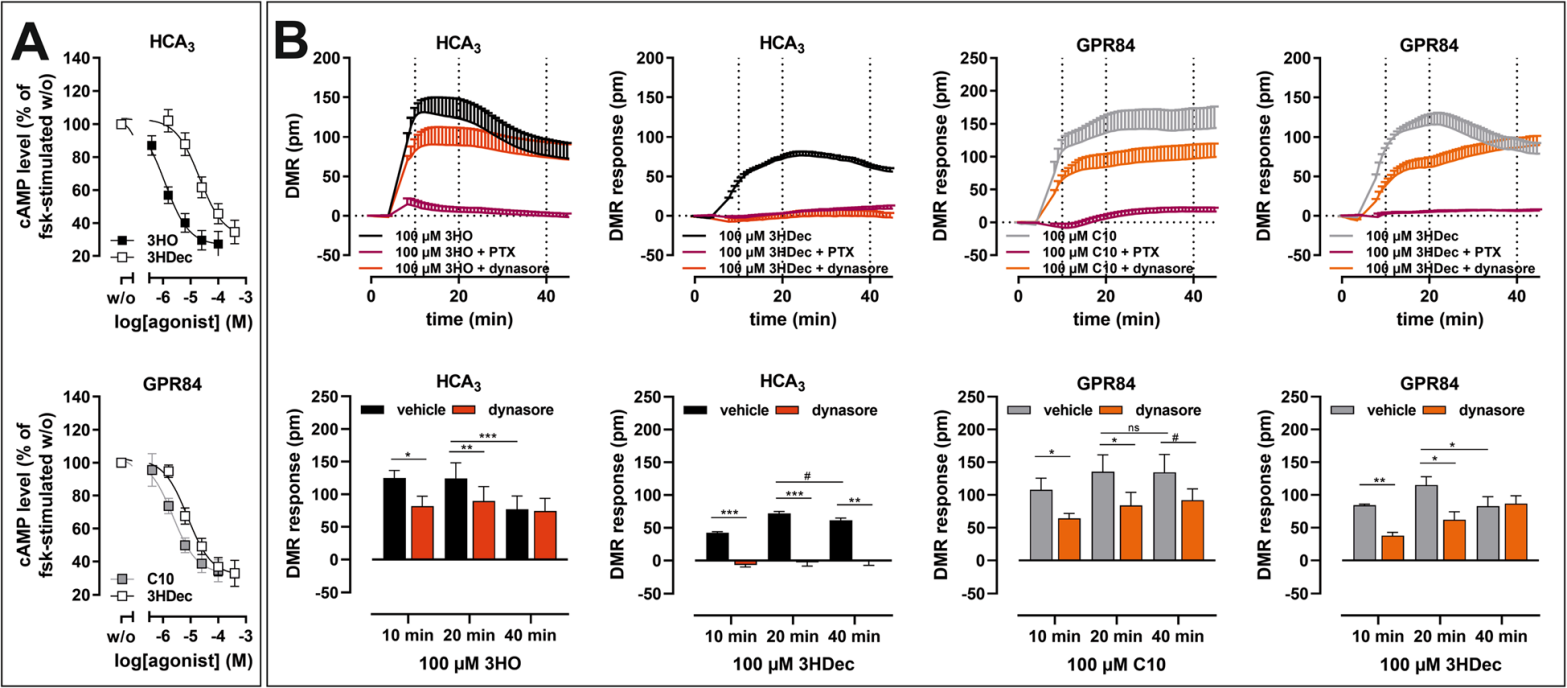
Differential agonist-dependent receptor activation kinetics and dynasore sensitivity of HCA_3_ and GPR84 DMR responses. CHO-K1 cells were transiently transfected with receptor constructs. **a** cAMP inhibition assays in presence of 2 μM forskolin (fsk) were performed which showed activation of HCA_3_ by 3-hydroxyoctanoic acid (3HO) and 3-hydroxydecanoic acid (3HDec). C10 and 3HDec activated GPR84. cAMP level of HCA_3_- or GPR84-transfected cells in absence of agonist is set 100%, respectively. **b** Transfected CHO-K1 cells were seeded in fibronectin-coated Epic plates and DMR responses were recorded. 3HO and 3HDec induced a pertussis toxin (PTX, 100 ng/ml)-sensitive DMR response in HCA_3_-, C10 and 3HDec in GPR84-expressing CHO-K1 cells. DMR responses in absence of dynasore showed distinct receptor activation kinetics for both, HCA_3_ and GPR84, when comparing the shown agonists, respectively. Dynasore (80 µM) diminished the 3HDec-induced DMR response of HCA_3_-expressing cells completely. Time points 10 min, 20 min and 40 min were used to generate bar graphs (concentration-response curves see Figures S2, S3) to highlight, that DMR responses of HCA_3_ to 3HO and GPR84 to 3HDec were affected by dynasore only at earlier time points. The DMR responses of HCA_3_ to 3HDec and of GPR84 to C10 were diminished over the whole time recorded. Shown is the agonist-induced wavelength shift in pm of three to five independent experiments, each carried out in triplicates as mean ± SEM. ns, not significant; ^#^*P* ≤ 0.1; * *P* ≤ 0.05; ** *P* ≤ 0.01

First, we confirmed that HCA_3_ and GPR84 induce cAMP inhibitory signaling in our experimental setup. Both, HCA_3_ and GPR84, exhibited increased basal activity in the G_i_ protein-mediated pathway, which was reflected in the reduced cAMP levels in CHO-K1 cells heterologously expressing the receptors compared to empty vector-transfected cells (Figure S1A). As expected, we also observed a concentration-dependent decrease of intracellular cAMP levels upon stimulation of HCA_3_ and GPR84 with their respective agonists (Fig. 1a, Table S2). Agonist stimulation usually results in receptor internalization reducing the receptor number at the cell surface (Figure S1B, β_2_-adrenergic receptor (ADBR2) and V2 vasopressin receptor (V2R)). However, only 3HO induced a significant reduction in cell surface expression of HCA_3_ (Figure S1B). These analyses confirmed previously published results on G-protein specificity and provided first evidences for agonist- and receptor-specific differences in the internalization of HCA_3_ and GPR84. Thus, we used the dynamic mass redistribution technology (DMR; Corning Epic System) to analyze the signaling kinetics of HCA_3_ and GPR84 in transiently transfected CHO-K1 cells.

### Differential activation kinetics of HCA_3_ and GPR84 upon agonist stimulation

The DMR technology provides the advantage of time-resolved recording (kinetics) of cellular responses independent of the activated signaling cascade. In DMR assays, HCA_3_ was also activated by 3HO and 3HDec but not by C10 (Figs. 1b, S2, S3, Table S2). In contrast, C10 and 3HDec but not 3HO activated GPR84 (Figs. 1b, S2, S3, Table S2). 100 ng/ml PTX treatment overnight completely abolished DMR responses of HCA_3_ and GPR84. Interestingly, we observed differences in receptor signaling kinetics. The maximum DMR response was reached faster when HCA_3_ was stimulated with 3HO compared to 3HDec (Fig. 1b). Moreover, the 3HDec-induced DMR response of GPR84 declined more rapidly compared to C10 (Fig. 1b). Despite equal PTX sensitivity, our DMR analyses revealed significant differences in agonist-induced signaling kinetics for both receptors, potentially due to different desensitization/internalization mechanisms.

### 3HDec-induced HCA_3_ DMR signal inhibited by dynasore

Responses recorded with the DMR technology reflect a sum of dynamic changes and signaling events induced by stimulation of a GPCR by its agonists, thus also including internalization and recruitment of signaling components. The neuropeptide Y receptor family is an example for receptors sharing agonists but exhibiting different internalization mechanisms upon activation [31]. We first used DMR assays to analyze whether HCA_3_ and GPR84 signaling is dependent on the small GTPase dyn-2, which is involved in the membrane scission step during endocytosis / internalization of some GPCRs and many other cellular processes [32, 33]. We applied the dyn inhibitor dynasore, a cell-permeable non-competitive inhibitor of the GTPase activity of dyn [34–36], in DMR analyses of GPR84 and HCA_3_ activation. We found that presence of dynasore differentially affected the DMR response of HCA_3_ and GPR84. The 3HO-induced HCA_3_ signal was reduced in presence of dynasore at earlier time points, whereas the 3HDec-induced DMR response of HCA_3_-transfected cells was completely abolished (Figs. 1b, S2, S3).

HCA_3_ is the evolutionarily youngest ortholog of the hydroxycarboxylic acid receptor (HCAR) family and only present in humans and apes [25]. We tested whether the observed differences for HCA_3_ activated by different agonists in the presence of dynasore is common in the HCAR family and thus evolutionarily conserved. We analyzed HCA_1_ and HCA_2_ DMR responses to different agonists in absence and presence of dynasore. Lactate, the endogenous agonist of HCA_1_, induced a DMR response, which was not inhibited but prolonged by dynasore (Figure S4A). A similar observation was made when HCA_1_ was stimulated with the surrogate agonist 3,5-dihydroxybenzoic acid (3,5-DHB) (Figure S4A). Similarly, the DMR responses of HCA_2_ to the endogenous agonist 3-hydroxybutyrate and the surrogate agonist monomethyl fumarate were sustained in presence of dynasore (Figure S4B). Therefore, the observed activation kinetics and dynasore-sensitivity of HCA_3_ are specific for this HCAR subtype. DMR responses of GPR84 were also affected by dynasore. The C10-induced signal was reduced in GPR84-transfected cells over the whole recording time whereas the 3HDec-induced signal was only affected at earlier time points (Figs. 1b, S2, S3).

In sum, our data highlights a differential dynasore-sensitivity of both receptors depending on the activating compound.

### Differential effects of the internalization inhibitors dynasore and sucrose on G_i_ protein- and ERK signaling of HCA_3_ and GPR84

**Fig. 2:**
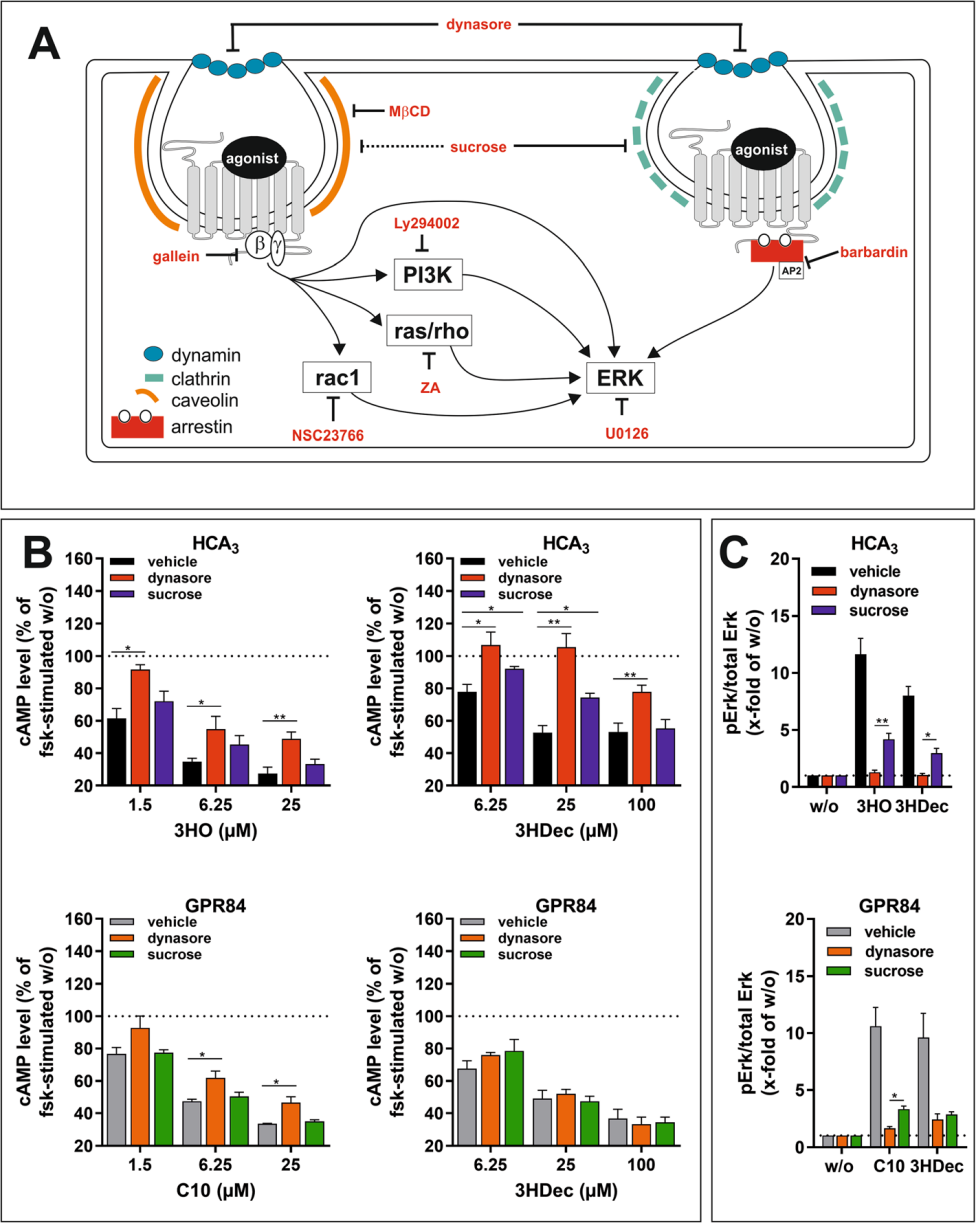
Effect of dynasore and sucrose on HCA_3_ and GPR84 cAMP inhibitory and ERK signaling. **a** Scheme summarizing all inhibitors and their targets used in the present study. **b, c** CHO-K1 cells were transiently transfected with HCA_3_ or GPR84. **b** 80 μM dynasore reduced cAMP inhibitory signaling of HCA_3_ upon 3HO and 3HDec and of GPR84 upon C10 but not 3HDec stimulation. Sucrose (0.4 M) significantly inhibited only the 3HDec-induced HCA_3_-mediated reduction of intracellular cAMP levels. cAMP level of HCA_3_- or GPR84-transfected cells in absence of agonist is set to 100%, respectively. **c** The agonist-induced phosphorylation of ERK1/2 was measured in absence and presence of dynasore or sucrose. All agonists induced an increase in pERK/total ERK levels upon stimulation of HCA_3_ and GPR84, respectively. Dynasore blocked the signal of 100 μM 3HO and 100 μM 3HDec completely and sucrose partially in HCA_3_–transfected cells. Dynasore did not fully block the signal of 100 μM C10 and 100 μM 3HDec in GPR84-transfected cells. The residual ERK signals of GPR84 in presence of 3HDec and dynasore or sucrose did not differ. pERK/total ERK of HCA_3_- or GPR84-transfected cells in absence of agonist is set 1. **b, c** Data is given as mean ± SEM of at least three independent experiments each carried out in triplicates. * *P* ≤ 0.05; ** *P* ≤ 0.01

Next, we asked whether the observed differences in agonist-induced DMR signals in the presence of dynasore are accompanied by differences in second messenger levels. In addition to dynasore, we used hyperosmolar sucrose, a non-specific inhibitor of receptor endocytosis, to test whether their presence results in prolonged HCA_3_- or GPR84-mediated cAMP inhibition (Fig. 2a, b) [37]. Hyperosmolar sucrose cannot be used in DMR assays because the induced cell shrinkage causes a constant shift in the reflected wavelength and destroys the assay window [38]. Hyperosmolar sucrose inhibits both, clathrin-mediated endocytosis and other endocytic routes, such as the caveolar pathway, but does not interfere with dyn-2 function or cAMP accumulation [39–41].

We pretreated CHO-K1 cells transiently transfected with HCA_3_ and GPR84 with 80 μM dynasore and 0.4 M sucrose for 30 min as previously described [39] and performed cAMP inhibition assays with the two respective agonists. In the presence of dynasore the 3HO-induced HCA_3_-mediated and the C10-induced GPR84-mediated reductions in cAMP levels were decreased (Fig. 2b, Table S3). Further, dynasore inhibited the 3HDec-induced reduction of cAMP levels in HCA_3_- but not in GPR84-transfected cells (Fig. 2b). Thus, the presence of dynasore did not prolong but inhibited the HCA_3_-mediated cAMP inhibitory signaling of all agonists tested. Dynasore inhibits dyn-2, which is not only a key protein of endocytosis but also of the intracellular membrane trafficking machinery [38]. Thus, the observed effects of dynasore on cAMP inhibitory signaling could be due to dyn-2-dependent trafficking of HCA_3_.

Moreover, we found that hypertonic sucrose significantly reduced the 3HDec-induced HCA_3_-mediated cAMP inhibition (Fig. 2b). No significant effect of sucrose on the GPR84-mediated inhibitory cAMP signal for either C10 or 3HDec was observed (Fig. 2b). Neither dynasore nor sucrose caused an effect in empty vector-transfected control cells (Figure S5).

Activation of both, HCA_3_ and GPR84, resulted in phosphorylation of extracellular-signal regulated kinase (ERK) (Figure S6A, Table S2). We did not observe calcium signals upon stimulation of either receptor (Figure S6B) which partially contrasts previous findings [42, 43]. However, the previously reported calcium signals in HCA_3_-transfected CHO-K1 cells have been reported at very high agonist concentrations, i.e. 100 μM of the synthetic agonist 1-isopropylbenzotriazole-5-carboxylic acid (IPBT5CA) [42], which has been reported to activate HCA_3_ with an EC_50_ value of 0.4 μM [44]. Additionally, Gaidarov et al. showed that GPR84 activation evoked calcium responses only in human macrophages but not in transfected HEK293 cells which is in line with our findings [43].

The presence of the MEK inhibitor U0126 (10 μM, Fig. 2a), which inhibits ERK signaling directly upstream of ERK [45], diminished the agonist-induced ERK phosphorylation of HCA_3_ and GPR84 completely (Figure S6C). Next, we analyzed the influence of 0.4 M sucrose and 80 μM dynasore on ERK signaling. Dynasore completely blocked the signal of HCA_3_ in response to both, 3HO and 3HDec, and sucrose inhibited it partially (Fig. 2c, Table S4). The C10-induced ERK signal of GPR84 was completely inhibited by the presence of dynasore but to a significantly lesser degree in the presence of sucrose (Fig. 2c). In contrast, dynasore and sucrose did not fully inhibit the ERK signal of GPR84 induced by 3HDec (Fig. 2c).

In summary, the ERK signal of both, HCA_3_ and GPR84, is dynasore- and to some extent sucrose-sensitive, and therefore internalization-dependent.

### Diminished signaling and altered subcellular distribution of HCA_3_ in the presence of dyn-2 mutants

**Fig. 3:**
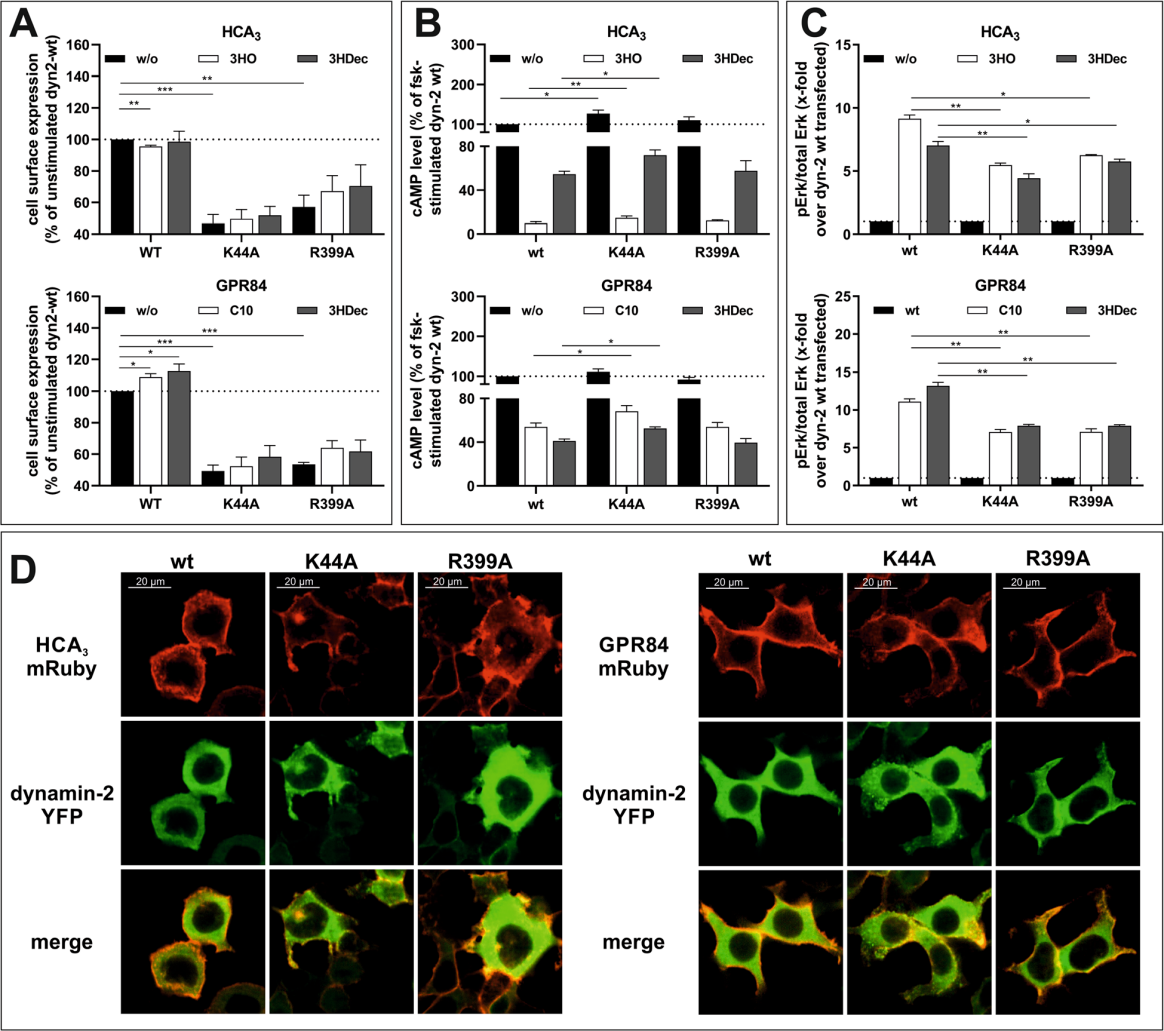
Effect of dyn-2 mutants on HCA_3_ and GPR84 cell surface expression, cAMP inhibitory signaling and ERK activation. **a-c** CHO-K1 cells were transiently co-transfected with HCA_3_ or GPR84 and dyn-2 wt, dyn-2 K44A or R399A mutants. **a** In comparison to dyn-2 wt co-transfected cells HCA_3_ and GPR84 cell surface expression was significantly reduced when K44A or R399A were co-transfected. **b** Basal activity of HCA_3_ but not GPR84 was diminished in presence of K44A. Agonist-induced (HCA_3_: 6.25 μM 3HO, 25 μM 3HDec; GPR84: 100 μM C10, 25 μM 3HDec) inhibition of forskolin-stimulated cAMP accumulation was reduced in presence of K44A compared to dyn-2 wt whereas R399A did not affect cAMP inhibitory signaling. **c** Agonist-induced increase of pERK/total ERK level of HCA_3_ (25 μM 3HO, 100 μM 3HDec) and GPR84 (25 μM C10, 25 μM 3HDec) was reduced in presence of K44A and R399A compared to dyn-2 wt. **a-c** Data is given as mean ± SEM of at least three independent experiments each carried out in triplicates. * *P* ≤ 0.05; ** *P* ≤ 0.01, *** *P* ≤ 0.001 (**d**) Images of HEK293-T cells transiently co-expressing HCA_3_-mRuby (red) or GPR84-mRuby and dyn-2-YFP variants (green). In presence of dyn-2 wt, HCA_3_ was detected intracellularly and at the plasma membrane where it co-localized with dyn-2 wt. In case of co-expression of HCA_3_ with the dyn-2 mutants K44A and R399A, co-localization was detected in perinuclear vesicles as well as certain areas at the plasma membrane. GPR84 was in presence of all dyn-2 variants found mostly at the plasma membrane

Since dynasore affected signal transduction of HCA_3_ and GPR84, we next co-transfected CHO-K1 cells with HCA_3_ or GPR84 and functionally altered dyn-2 variants to further investigate the role of dyn-2 for receptor trafficking, signaling and internalization. In the K44A dyn-2 mutant, the GTP-binding ability is impaired causing lower GTP hydrolysis, which blocks the coated vesicle formation without affecting the coat assembly and invagination [46]. Moreover, we used the R399A dyn-2 variant with an impaired self-assembly and membrane localization [47]. Using ELISA, we found that cell surface expression of HCA_3_ and GPR84 was reduced in the presence of either dyn-2 mutant (Fig. 3a). No internalization of HCA_3_ upon stimulation with 3HO was detected when dyn-2 K44A or R399A were co-transfected (Fig. 3a).

Analyses of co-expression of HCA_3_ with dyn-2 wt and mutants in cAMP inhibition assays revealed significantly increased basal cAMP levels in the presence of K44A indicating a reduced basal activity of HCA_3_ (Fig. 3b). These higher cAMP levels in the presence of K44A persisted when HCA_3_ was activated with 3HO or 3HDec, whereas the presence of R399A did not cause altered cAMP signaling (Fig. 3b). In contrast, co-expression of GPR84 with dyn-2 K44A compared to dyn-2 wt did not affect basal activity of GPR84, but reduced receptor activation by both C10 and 3HDec (Fig. 3b). Moreover, we found that agonist-induced activation of ERK by HCA_3_ and GPR84 was significantly reduced in the presence of both, K44A and R399A, compared to dyn-2 wt (Fig. 3c).

Next, we used HEK293-T cells due to their better suitability for image analyses and at first confirmed that HCA_3_ and GPR84 also exhibit a basal activity in HEK293-T cells (Figure S7A). In addition, we showed that in HEK293-T cells, similar to our observations in CHO-K1 cells, a concentration-dependent decrease in intracellular cAMP levels was observed upon stimulation of HCA_3_ with 3HO and 3HDec as well as of GPR84 with C10 and 3HDec (Figure S7A). Further, also in HEK293-T cells only stimulation with 3HO induced a significant reduction in cell surface expression levels of HCA_3_ (Figure S7B). However, using ELISA we found that in contrast to CHO-K1 cells, in HEK293-T cells only HCA_3_ but not GPR84 cell surface expression was significantly reduced in the presence of either dyn-2 mutant (Figure S7C).

Next, we transiently co-transfected HEK293-T cells with mRuby-tagged HCA_3_ or mRuby-tagged GPR84 and YFP-tagged dyn-2 variants for subcellular localization analyses. HCA_3_ was expressed at the plasma membrane, where it co-localized with dyn-2 wt (Fig. 3d). In case of co-expression of HCA_3_ with either of the dyn-2 mutants K44A or R399A, co-localization was found in perinuclear vesicles as well as in certain areas at the plasma membrane indicating that expression of dyn-2 mutants impairs HCA_3_ trafficking and thereby reduces its cell surface expression (Fig. 3d). Substantiating our findings from ELISA analyses, we found that in HEK293-T cells GPR84 plasma membrane expression was not reduced in presence of dyn-2 mutants and GPR84 was not found co-localized with either dyn-2 mutant in perinuclear vesicles (Figs. 3d, S7C).

Finally, we performed ELISA analyses in CHO-K1 cells in the presence of dynasore. Cell surface expression of ADRB2 and V2R, serving as controls, were reduced by only 15%, while HCA_3_ and GPR84 cell surface expression levels were decreased by about 50 and 30%, respectively (Figure S8).

### No effect of methyl-β-cyclodextrin (MβCD), an inhibitor of caveolar endocytosis, on 3HDec-induced GPR84 signaling

**Fig. 4:**
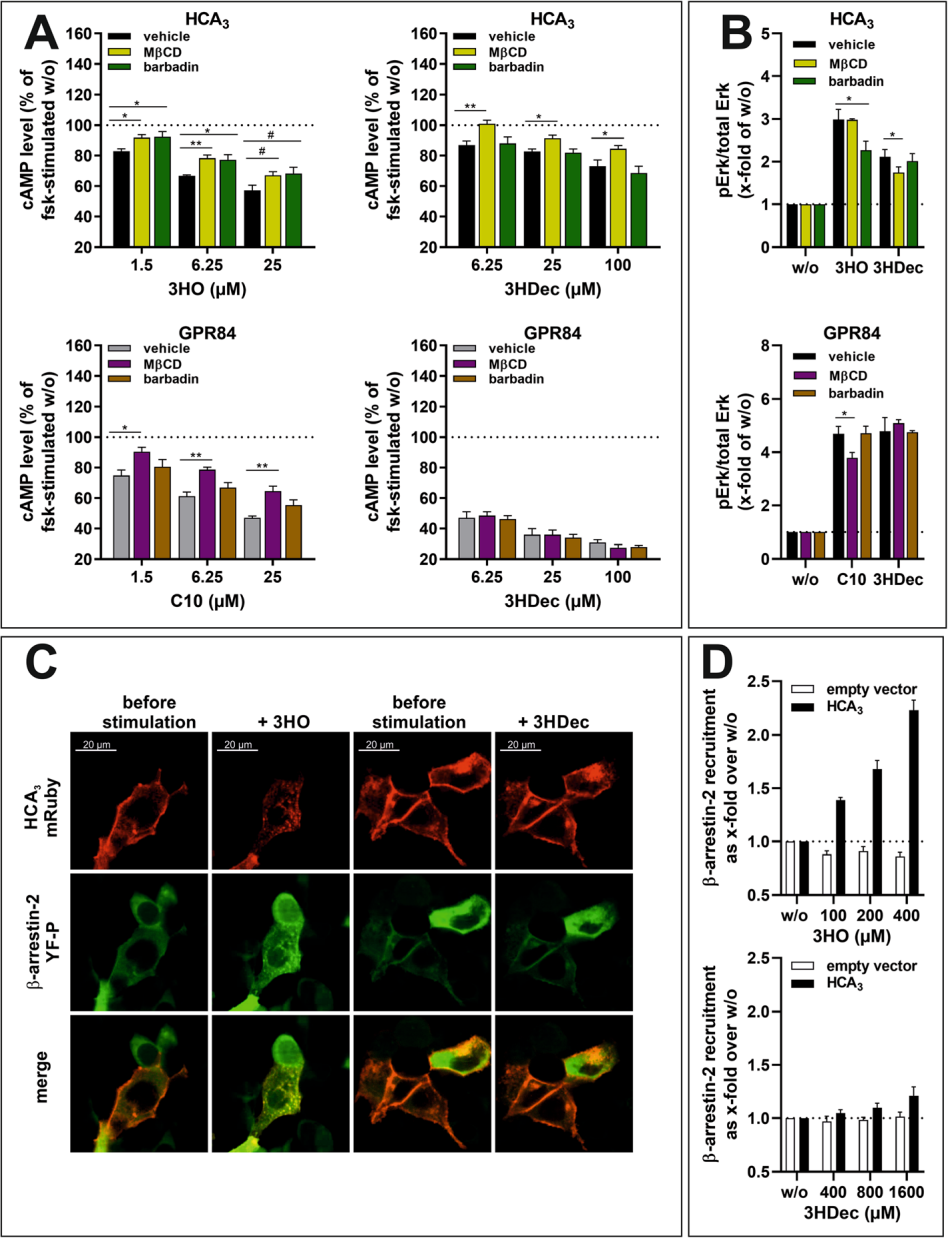
Role of β-arrestin-2 for HCA_3_ and GPR84 signaling and effect of methyl-β-cyclodextrin (MβCD). **a, b** CHO-K1 cells were transiently transfected with HCA_3_ or GPR84. **a** MβCD inhibited both, the 3HO- and 3HDec-induced reduction of forskolin (fsk)-induced cAMP levels in HCA_3_-transfected cells. For GPR84, only the C10-induced but not the 3HDec-induced decrease in cAMP was inhibited. Barbardin (100 µM) inhibited only the 3HO-induced HCA_3_-mediated reduction of cAMP levels. cAMP levels of HCA_3_- or GPR84-transfected cells in absence of agonist are set 100%, respectively. **b** 3 mM MβCD did not affect HCA_3_-mediated activation of ERK by 3HO, but caused a decrease of the signal induced by 100 μM 3HDec. Presence of MβCD caused a decrease in C10-induced GPR84-mediated ERK activation and had no effect on the 3HDec-induced ERK activation. The HCA_3_-mediated activation of ERK by 3HO, but not 3HDec, was inhibited in presence of barbardin. Barbardin had no effect on the GPR84-mediated activation of ERK by 3HDec and C10. pERK/total ERK of HCA_3_- or GPR84-transfected cells in absence of agonist is set 1, respectively. **c** Live-cell images of HEK293-T cells co-expressing HCA_3_-mRuby (red) and β-arrestin-2-YFP (green) were acquired before stimulation and 30 min post-stimulation with 100 μM 3HO or 100 μM 3HDec. **d** HEK293-T cells stably expressing β-arrestin-2-EA cells transiently transfected with HCA_3_ were stimulated with 3HO and 3HDec. Quantification of β-arrestin-2 recruitment using the PathHunter β-arrestin assay (Eurofins DiscoverX) showed recruitment of β-arrestin-2 by HCA_3_ following 3HO but not 3HDec stimulation. Luminescence of HCA_3_ or empty vector transfected cells in absence of agonist is set 1, respectively. **a, b, d** Data is given as mean ± SEM of at least three independent experiments each carried out in triplicates. ^#^*P* ≤ 0.1* *P* ≤ 0.05; ** *P* ≤ 0.01

The best-characterized internalization routes of GPCRs are clathrin-mediated or caveolae-dependent pathways [37]. Dynasore alone does not allow discrimination of the two pathways because both depend on dyn-2. However, caveolar endocytosis is sensitive to cholesterol depletion by MβCD and previous studies showed that 3 mM of MβCD selectively inhibit caveolar endocytosis of ADRB2 and the dopamine D2 receptor [37]. Thus, we tested the impact of MβCD on HCA_3_ and GPR84 signaling to reveal potential differences in caveolar endocytic processes. MβCD abolished the 3HO-induced HCA_3_-mediated and C10-induced GPR84-mediated reduction in cAMP levels (Fig. 4a, Table S3). However, MβCD inhibited the 3HDec-induced reduction of cAMP only in HCA_3_ but not in GPR84-transfected CHO-K1 cells (Fig. 4a). MβCD did not cause an effect in empty vector-transfected control cells (Figure S5). Further, the 3HDec- but not the 3HO-induced ERK activation in HCA_3_-expressing cells was reduced in the presence of MβCD (Fig. 4b). On the contrary, the presence of MβCD inhibited the ERK signal of GPR84 induced by C10 but did not diminish the GPR84-mediated activation of ERK by 3HDec (Fig. 4b, Table S4).

### β-arrestin-2 recruitment upon activation of HCA_3_ by 3HO but not 3HDec

Several adaptor and accessory proteins are involved in the process of GPCR internalization, including arrestins and the adaptor protein complex 2 (AP2) with the subunit β_2_-adaptin [48]. Therefore, we analyzed whether β-arrestin-2 is involved in mediating signal transduction of HCA_3_ and GPR84. We used 100 μM barbardin, which is a selective arrestin/β_2_-adaptin inhibitor that blocks agonist-promoted arrestin-dependent and clathrin-mediated endocytosis, but does not interfere with the translocation of arrestin to the receptor or with the interaction of AP2 with other components of the endocytic machinery [49].

We found that barbardin diminished only the 3HO-induced HCA_3_-mediated cAMP inhibitory signaling but not the 3HDec-induced HCA_3_-dependent or the GPR84-mediated signaling (Fig. 4a, Table S3). No effect of barbardin was observed in empty vector-transfected control cells (Figure S5). Barbardin treatment only inhibited the HCA_3_-mediated ERK signal upon 3HO stimulation, but had no effect on the 3HDec-induced HCA_3_-mediated ERK signal or the GPR84-mediated ERK signal induced by either agonist (Fig. 4b, Table S4).

This data suggests that arrestin recruitment only occurs when HCA_3_ is activated by 3HO. To further substantiate this finding, we set out for co-localization analyses of mRuby-tagged HCA_3_ and YFP-tagged β-arrestin-2. We used fluorescence confocal microscopy of living HEK293-T cells to analyze the subcellular distribution of HCA_3_ and β-arrestin-2 before and after stimulation with 3HO or 3HDec, respectively. Formation of internalized vesicles, where HCA_3_ and β-arrestin-2 are co-localized, was observed following incubation with 3HO, but not with 3HDec. When stimulated with 3HDec, HCA_3_ remained localized mostly at the plasma membrane (Fig. 4c). Finally, we used the PathHunter β-Arrestin assay (Eurofins DiscoverX) in HEK293-T cells stably expressing β-arrestin-2-EA to quantify 3HO and 3HDec-induced β-arrestin-2 recruitment of HCA_3_. Again, we found that activation of HCA_3_ by 3HO but not 3HDec leads to β-arrestin-2 recruitment (Fig. 4d).

Next, we used the HEK293-T cells stably expressing β-arrestin-2-EA to support our observation that GPR84 does not interact with β-arrestin-2 upon activation with C10 and 3HDec. Activation of GPR84 by neither C10 nor 3HDec induced a significant recruitment of β-arrestin-2 in HEK293-T cells as analyzed using both, the PathHunter β-Arrestin assay as well as co-localization analyses of mRuby-tagged GPR84 and YFP-tagged β-arrestin-2 (Figures S7D, S7E).

Finally, we tested whether barbardin affects cell surface expression of HCA_3_ and GPR84. We found that cell surface expression of ADRB2 and V2R, previously shown to internalize via β-arrestin-dependent and clathrin-mediated endocytosis, was reduced by about 15% in the presence of barbardin (Figure S8) [49]. No effect of barbardin on cell surface expression of GPR84 was observed, but a reduction for HCA_3_ cell surface expression by about 15% (Figure S8).

### Activation of HCA_3_ by LAB-derived metabolites caused β-arrestin-2 recruitment

Recently, we analyzed the evolutionary history of HCA_3_ and identified metabolites of lactic acid bacteria (LAB) as highly potent agonists [25]. This discovery prompted us to ask the following questions: First, is it possible to determine structurally relevant residues that could help to understand the different trafficking of HCA_3_ compared to HCA_1_ and HCA_2_ (Figs. 1, S4)? Second, are LAB-derived agonists equally distinguishable, like 3HO and 3HDec, when comparing their DMR response in the presence of dynasore? To answer the first question, we analyzed the gorilla and orangutan HCA_3_ orthologs in DMR assays and found that Tyr^86^ and Trp^142^ might be indirectly or directly involved in the interaction of HCA_3_ with dyn-2 (Supplementary results and discussion, Figure S9). To answer the second question, we performed DMR analyses with 4 μM D-phenyllactic acid (D-PLA), 4 μM indole 3-lactic acid (ILA), 250 μM D-phenylalanine (D-Phe), and 400 μM L-phenyllactic acid (L-PLA) on human, gorilla and orangutan HCA_3_ (Figure S9). These HCA_3_ agonists did not activate GPR84 (Figure S10A). Our analyses showed that dynasore affected the DMR-response of human and gorilla HCA_3_ to D-Phe and L-PLA in a similar manner like that to 3HDec, whereas the DMR response of D-PLA and ILA was similarly affected like the one to 3HO (Figure S9). At last, we performed β-arrestin-2 recruitment assays and found that activation of human HCA_3_ by both agonists, D-PLA and ILA, indeed caused recruitment of β-arrestin-2 whereas this was not the case for D-Phe (Figure S10B).

### Gβγ-dependent HCA_3_ signaling and internalization

**Fig. 5:**
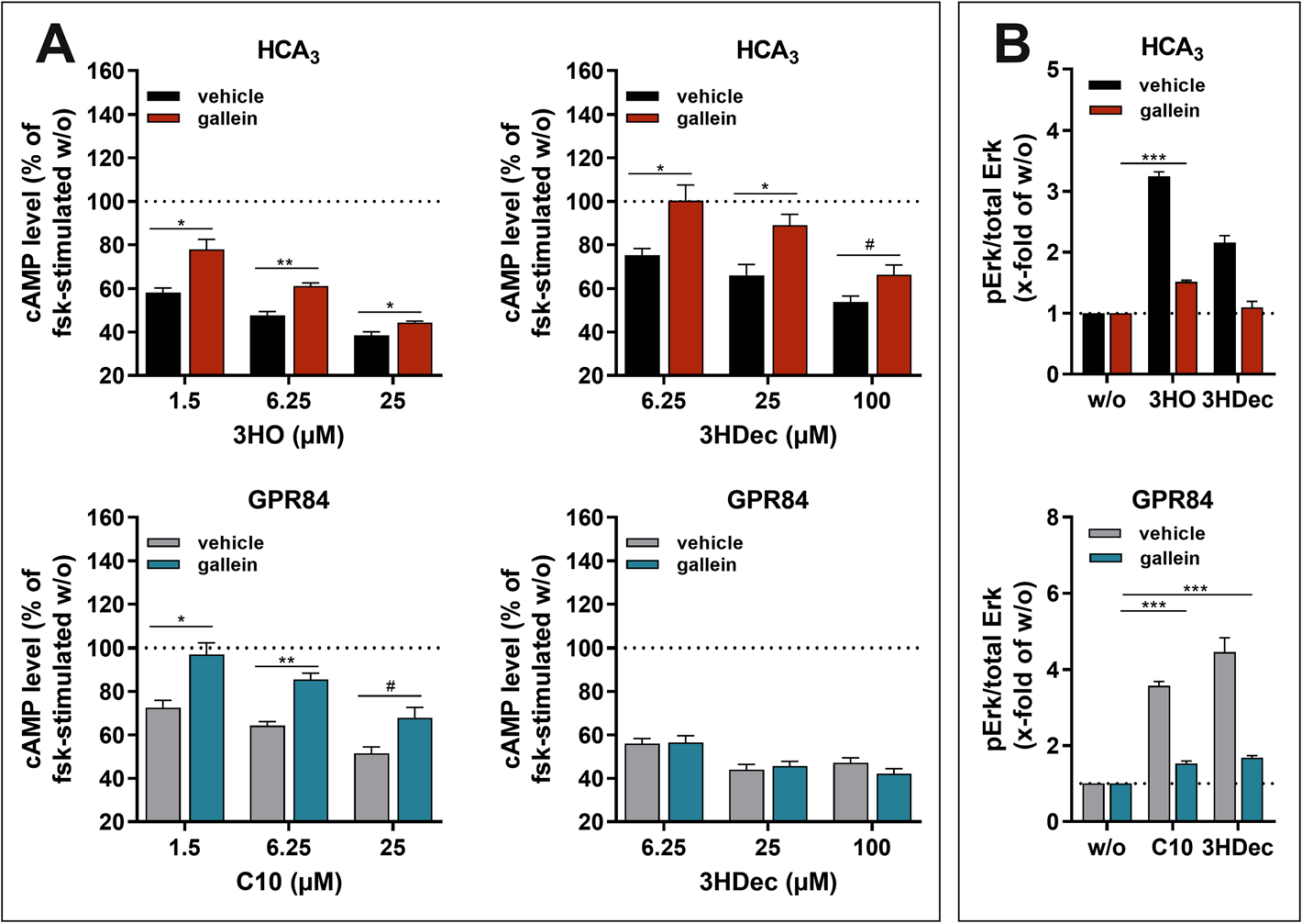
Effect of gallein, an inhibitor of Gβγ subunits, on agonist-induced reduction of cAMP levels and ERK activation of HCA_3_ and GPR84. CHO-K1 cells were transiently transfected with HCA_3_ or GPR84. **a** The HCA_3_-mediated reduction of forskolin (fsk)-induced cAMP levels induced by both, 3HO and 3HDec, was significantly diminished in presence of 50 μM gallein. The GPR84-induced decrease in cAMP levels in presence of gallein was only reduced in case of activation by C10 but not 3HDec. cAMP level of HCA_3_- or GPR84-transfected cells in absence of agonist is set 100%, respectively. **b** Gallein inhibited the 3HDec-induced HCA_3_-mediated increase in pERK/total ERK levels completely but the 3HO-induced increase only partially. GPR84-mediated activation of ERK by both, C10 and 3HDec, was equally diminished in presence of gallein. pERK/total ERK of HCA_3_- or GPR84-transfected cells in absence of agonist is set 1, respectively. **a, b** Data is given as mean ± SEM of at least three independent experiments each carried out in triplicates. * *P* ≤ 0.05; ** *P* ≤ 0.01; *** *P* ≤ 0.001

Gβγ subunits have been shown to interact with dyn, modulate its activity and thereby influence receptor internalization as well as trafficking [50]. Thus, we performed ELISA analyses to test whether gallein, an inhibitor of Gβγ signaling, interferes with cell surface expression of HCA_3_ and GPR84 [51]. We found that 50 μM gallein reduces HCA_3_ cell surface expression by about 20%, whereas it does not affect GPR84, ADRB2 and V2R cell surface expression, thus indicating that Gβγ subunits are involved in HCA_3_ trafficking (Figure S8). Further, Gβγ subunits modulate many effectors including adenylyl cyclase isoforms and ERK [52]. Hence, we analyzed the effect of gallein on both, cAMP inhibitory signaling and ERK activation [51]. The cAMP inhibitory response of HCA_3_ to both agonists, 3HO and 3HDec, was inhibited by gallein (Fig. 5a, Table S3). In contrast, presence of gallein diminished only the C10-induced but not the 3HDec-induced reduction of cAMP in GPR84-transfected CHO-K1 cells (Fig. 5a, Table S3). Gallein caused no effect in empty vector-transfected control cells (Figure S5).

ERK analyses revealed that the 3HDec-induced signal of HCA_3_ expressing cells was completely lost in the presence of gallein, whereas ERK activation was still detectable for 3HO (Fig. 5b, Table S4). Presence of gallein caused a reduction but did not completely abolish the GPR84-mediated ERK activation by C10 and 3HDec, which indicates that this ERK signal is partially Gβγ subunit-independent (Fig. 5b, Table S4).

### PI3K, ras/rho and rac1 involved in ERK activation downstream of HCA_3_

**Fig. 6:**
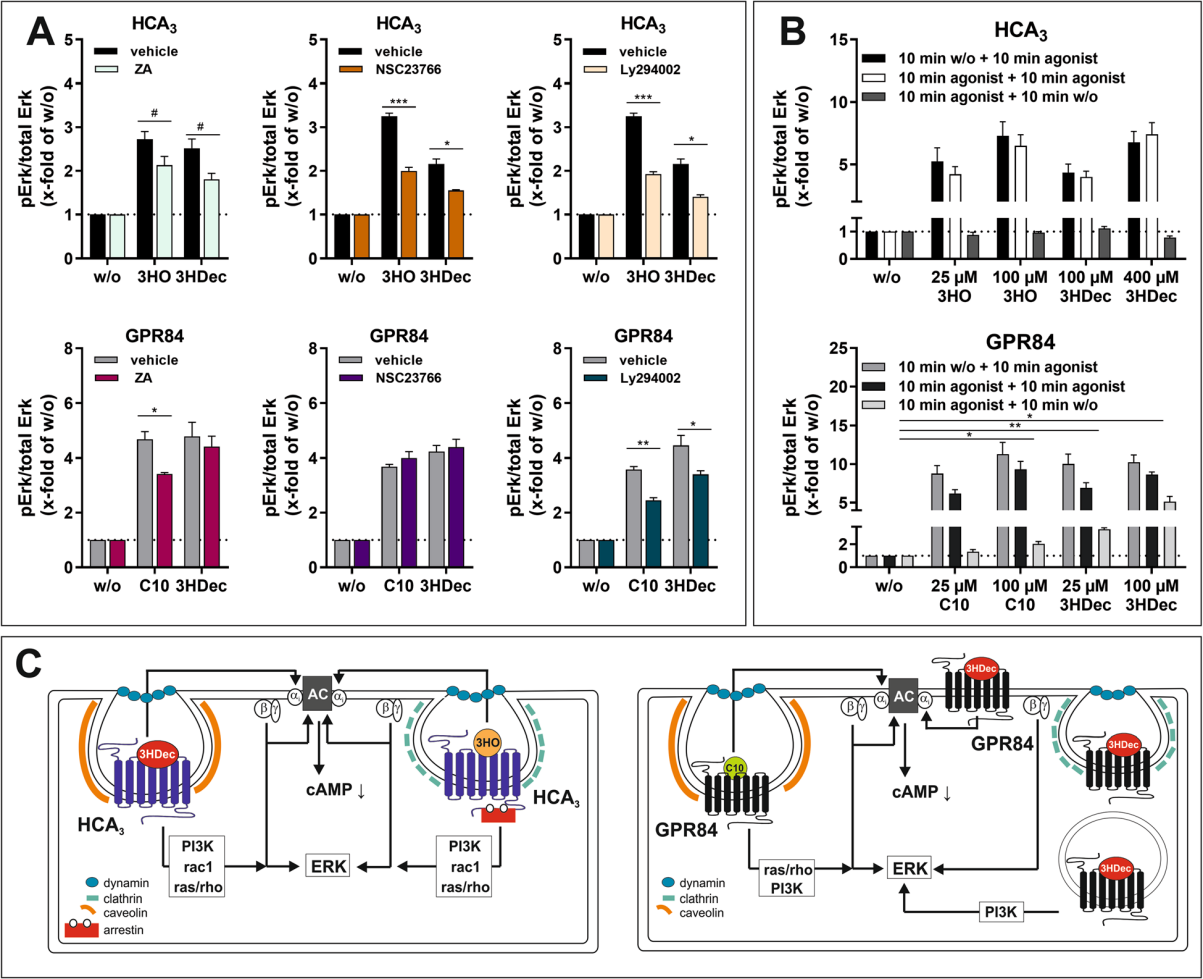
Components involved in HCA_3_ and GPR84 signal transduction. **a, b** Agonist-induced phosphorylation of endogenous ERK1/2 in cellular lysates of HCA_3_ or GPR84 transfected CHO-K1 cells in absence and presence of 25 μM ZA (zoledronic acid - inhibitor of ras/rho), 100 μM NSC23766 (inhibitor of rac1) and 25 μM Ly294002 (inhibitor of PI3K) was determined. **a** ZA, NSC23766 and Ly294002 partially inhibited the HCA_3_-induced ERK activation of both agonists. ZA and Ly 294,002 caused a significant reduction of the GPR84-mediated ERK activation by C10, whereas the ERK activation by 3HDec was only affected by presence of Ly294002. NSC23766 did not inhibit the GPR84-induced activation of ERK by either agonist. **b** Both, the 3HO- and 3HDec-induced ERK activation of HCA_3_ did not persist upon removal of agonist. The GPR84-mediated activation of ERK by 3HDec persisted, whereas the C10-induced activation was almost completely diminished 10 min past agonist removal. **a, b** pERK/total ERK of HCA_3_- or GPR84-transfected cells in absence of agonist is set 1, respectively. Data is given as mean ± SEM of at least three independent experiments each carried out in triplicates. ^#^*P* ≤ 0.1; * *P* ≤ 0.05; ** *P* ≤ 0.01; *** *P* ≤ 0.001. **c** 3HO- and 3HDec-induced cAMP inhibitory signaling of HCA_3_ was dependent on Gαi, Gβγ subunits and dyn (internalization). Signaling components involved in HCA_3_-mediated ERK activation by 3HO an 3HDec included Gβγ subunits, PI3K, rac1 and ras/rho. HCA_3_ activation by 3HO led to β-arrestin-2 recruitment, which was not the case for 3HDec. ERK signaling of HCA_3_ by 3HO involved clathrin and by 3HDec caveolin. GPR84 activation by C10 was dependent on Gαi, Gβγ subunits, dyn (internalization), caveolin, ras/rho and PI3K. In contrast, 3HDec-induced cAMP inhibitory signaling was not dependent on Gβγ subunits, dyn, caveolin or clathrin, thus internalization. ERK activation induced by GPR84 upon 3HDec stimulation persisted upon agonist removal and involved PI3K

As shown above, HCA_3_ trafficking and signaling is dependent on Gβγ subunits. Since other downstream effectors of Gβγ subunits include PI3-kinase γ and rac1, which are crucial for chemotaxis in leukocytes, we next analyzed the effect of inhibitors of ras/rho, rac1 and PI3K on the agonist-induced ERK activation (Fig. 2a) [51]. Zoledronic acid (ZA), an inhibitor of ras/rho, reduced the HCA_3_-mediated ERK activation by both HCA_3_ agonists and the C10- but not the 3HDec-induced GPR84-mediated signal (Fig. 6a, Table S4). Presence of 100 μM NSC23766, an inhibitor of rac1, inhibited both, the 3HO- and the 3HDec-induced HCA_3_-mediated ERK signal, but showed no effect on the GPR84-mediated signal, independent of the agonist (Fig. 6a, Table S4). LY294002 (25 µM), a PI3K inhibitor, caused a reduction of all agonist-induced HCA_3_- and GPR84-mediated ERK signals (Fig. 6a, Table S4).

### Sustained GPR84-mediated ERK activation by 3HDec after agonist washout

Finally, we performed agonist-washout experiments to test whether any of the observed agonist-dependent differences observed for HCA_3_ and GPR84 can be explained by persistent signaling, i.e. ERK activation when the respective agonist is removed. We found that 10 min after agonist removal ERK activation was completely abolished to basal levels in HCA_3_–transfected cells independent of the agonist (Fig. 6b). In contrast, in GPR84-expressing cells, 10 min after 3HDec removal, we still detected a significant activation of ERK for both concentrations, 25 μM (3-fold over basal) and 100 μM (5-fold over basal) (Fig. 6b). For C10, a significant residual ERK signal was only detectable for 100 μM (2-fold over basal) (Fig. 6b). This data suggests that 3HDec induces persistent signaling of GPR84.

In summary, our functional analyses revealed that β-arrestin-2-dependence is the major difference in 3HO- versus 3HDec-induced HCA_3_ signaling (Fig. 6c). For GPR84, we showed that C10 induces a signal depending on Gβγ, dyn (internalization), caveolin, ras/rho and PI3K. In contrast, cAMP inhibitory signaling of GPR84 upon stimulation with 3HDec is independent of Gβγ, dyn, caveolin or clathrin and thus internalization (Fig. 6c). Comparing signaling components involved upon stimulation of HCA_3_ and GPR84 with 3HDec, we found that while the HCA_3_-mediated ERK activation is dependent on caveolin, PI3K, rac1 and ras/rho, the GPR84-mediated ERK activation only involves PI3K (Figs. 4b, 6a, c). Moreover, dynasore, sucrose, MβCD and gallein affect cAMP inhibitory signaling of HCA_3_ but not of GPR84 suggesting an involvement of internalization as well as Gβγ subunits in HCA_3_-mediated signaling upon stimulation with 3HDec which is not the case for GPR84 (Figs. 2b, 4a, 5a).

For GPR84 it has recently been demonstrated that different surrogate ligands differentially induce chemotaxis in macrophages while similarly enhancing the levels of phagocytosis [53]. However, for HCA_3_ no information is available analyzing the biological consequences and potential differences upon activation of HCA_3_ by different agonists. Here, we used in vitro spheroid models to analyze whether the agonists differentially influence growth or density.

### CHO-K1 cell spheroid formation affected by HCA_3_

**Fig. 7:**
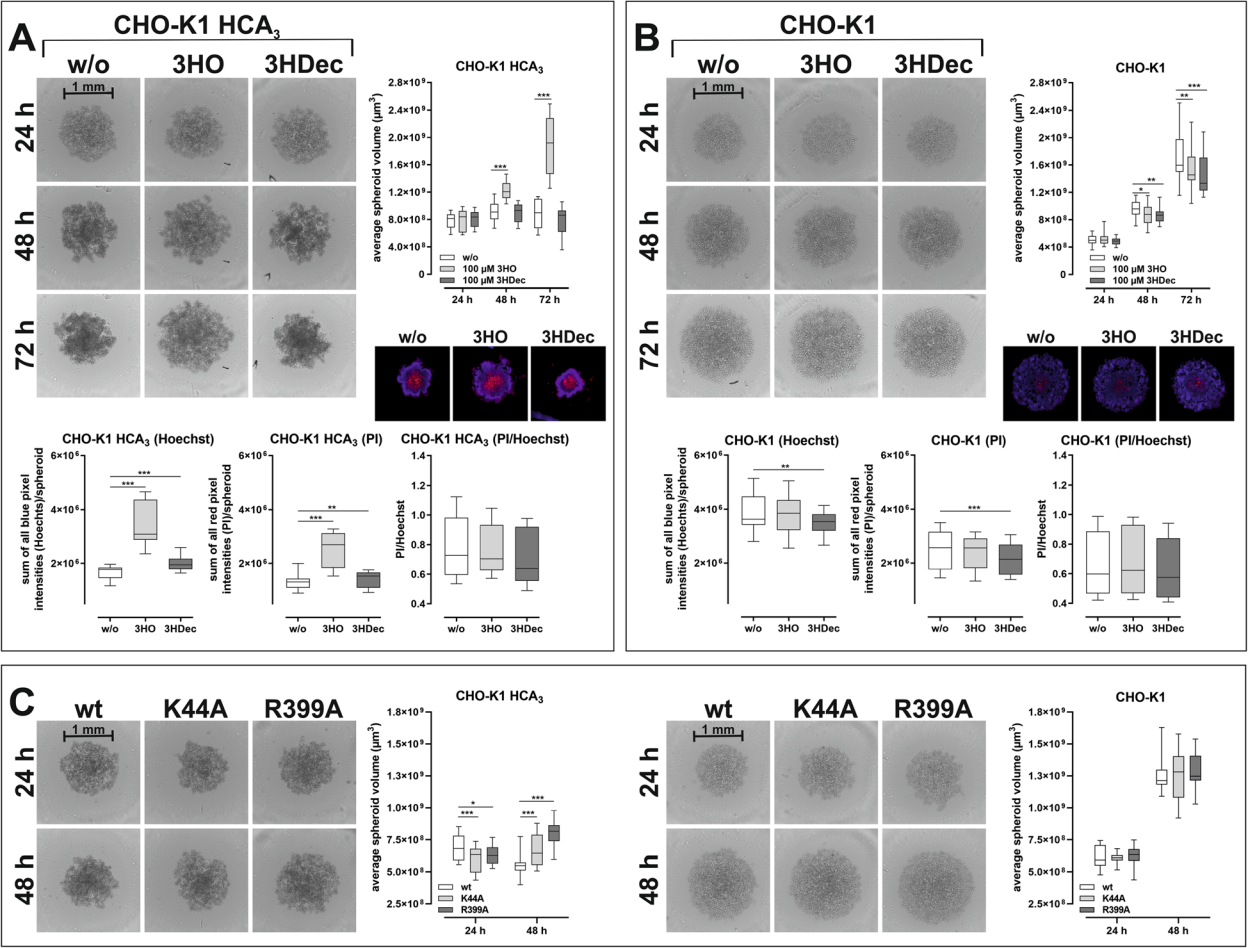
Growth and density of HCA_3_-expressing CHO-K1 spheroids. CHO-K1 cells stably expressing HCA_3_ (Eurofins Discover X) and CHO-K1 control cells were cultured in 96-well ultra-low attachment plates in absence and presence of 3HO and 3HDec (**a, b**) or after transfection with dyn-2 wt, K44A or R399A for the indicated time (**c**). Acquisition of images and analyses of the average spheroid volume was performed every 24 h using a Celigo Image Cytometer. **a** Presence of 3HO, but not 3HDec, caused significantly larger HCA_3_-expressing CHO-K1 spheroids. Total (Hoechst 33342 staining) and dead cell (propidium iodide (PI) staining) pixel counts for spheroids in presence of 3HO and 3HDec were significantly higher than in absence of HCA_3_ agonists but the ratio of dead cells to total cells did not differ. **b** CHO-K1 control cells spheroids were smaller in presence of 3HO or 3HDec. Total (Hoechst 33342 staining) and dead cell (propidium iodide (PI) staining) pixel counts for spheroids were significantly lower in presence of 3HDec. The ratio of dead cells to total cells did not differ. **c** The size of HCA_3_-expressing CHO-K1 spheroids was differentially affected by the presence of dyn-2 mutants whereas no effect was observed on spheroid growth of CHO-K1 control cells. **a-c** Data is shown as average spheroid volume in μm^3^ (box and whiskers min to max of *n* = 4 independent experiments, each carried out in 6 replicates). ^#^*P* ≤ 0.1; * *P* ≤ 0.05; ** *P* ≤ 0.01; *** *P* ≤ 0.001

First, we used a stably HCA_3_-expressing CHO-K1 cell line and analyzed the effect of 3HO and 3HDec on spheroid formation, structure and growth in comparison to a wt CHO-K1 cell line by culturing both in ultra-low attachment (ULA) plates. The spheroids were monitored for 72 h using the Celigo Image Cytometer. Three dimensional cell culture models mimic the physiological state regarding cell-cell contacts and nutrient gradients more closely than 2D cultures [54]. We observed that spheroids of HCA_3_-expressing CHO-K1 cells become denser over time in comparison to those of CHO-K1 cells that do not express the receptor (Fig. 7a, b). We attribute this observation to the fact that HCA_3_ exhibits basal activity (Figure S1A). On the contrary, activation of HCA_3_ by 3HO resulted in significantly larger but less dense spheroids compared to unstimulated HCA_3_-expressing CHO-K1 cells, whereas 3HDec did not have this effect (Fig. 7a). To substantiate this further, we stained the spheroids with Hoechst 33342 (blue) for total cell count and PI (red) for dead cell count. We found that blue and red pixel intensities for both, 3HO- and 3HDec-stimulated HCA_3_-expressing spheroids, were higher compared to unstimulated (Fig. 7a). However, the ratio of dead cells to total cells (PI/Hoechst) did not significantly differ between the different treatments. This again suggests that HCA_3_ activation by 3HO influences cell-cell adhesion and not proliferation or cell death (Fig. 7a). Both agonists did not have this effect in wt CHO-K1 cells (Fig. 7b).

Since HCA_3_ did not only exhibit basal activity but also dyn-2-dependent trafficking and signaling (Figs. 3, S8), we tested whether the mutants affected cell-cell adhesion in our spheroid model. We transfected the stably HCA_3_-expressing CHO-K1 cell line with either dyn-2 wt or one of the aforementioned mutants and cultured them for 48 h to allow spheroid formation. We found that the presence of dyn-mutants caused HCA_3_-expressing spheroids to be smaller 24 h post-seeding, but less dense 48 h post-seeding, whereas no such effects of the dyn-2 mutants were observed in CHO-K1 cells that did not express HCA_3_ (Fig. 7c).

In summary, these observations suggest that HCA_3_ exhibits a basal interaction with dyn-2, which mediates increased cell-cell adhesion and is interrupted upon activation with 3HO accompanied by β-arrestin-2-recruitment. Thus, both β-arrestin-2 recruitment and presence of dyn mutants result in less dense spheroids (Fig. 7).

### 3HO influence on spheroid density of the endogenously HCA_3_ expressing esophageal carcinoma cell line Colo680N

**Fig. 8:**
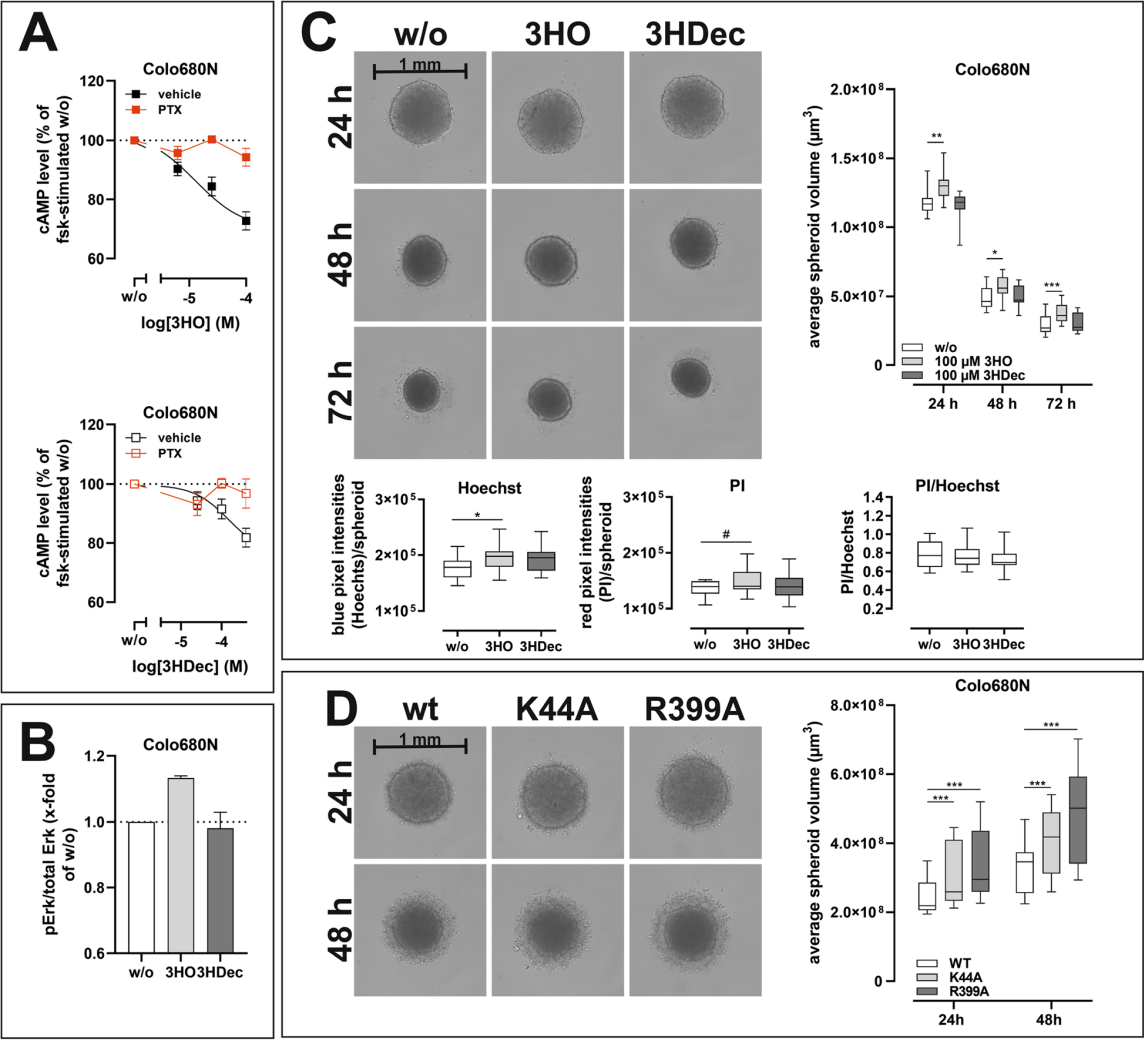
Spheroid density of the HCA_3_ expressing esophageal squamous cell carcinoma cell line Colo680N affected by HCA_3_ agonists and dyn mutants. Colo680N cells express HCA_3_. **a** cAMP inhibitory response induced by the HCA_3_ agonists 3HO and 3HDec was detected and sensitive to PTX. **b** Only 25 μM 3HO but not 100 μM 3HDec induced an ERK activation in Colo680N cells. **c, d** Colo680N cells were cultured in 96-well ultra-low attachment plates (Nexcelom) in absence and presence of 3HO and 3HDec (**c**) or after transfection with dyn-2 wt, K44A or R399A (**d**) for the indicated time. Acquisition of images and analyses of the average spheroid volume was performed every 24 h using a Celigo Image Cytometer. **c** Presence of 3HO, but not 3HDec, caused significantly larger Colo680N spheroids. Hoechst 33342 (total number of cells) and propidium iodide (PI, dead cells) staining revealed higher pixel counts for spheroids in presence of 3HO but the ratio of dead cells to total cells did not differ. **d** The size of Colo680N spheroids was affected by the presence of dyn-2 mutants K44A and R399A. **c, d** Data is shown as average spheroid volume in μm^3^ (box and whiskers min to max of *n* = 3 independent experiments, each carried out in 8 replicates). ^#^*P* ≤ 0.1; * *P* ≤ 0.05; ** *P* ≤ 0.01; *** *P* ≤ 0.001

We tested whether similar effects on spheroid formation can be observed in an endogenously HCA_3_-expressing cell line. We confirmed presence of HCA_3_ by functional characterization of the Colo680N cell line. We performed cAMP inhibitory assays and found PTX-sensitive activation by 3HO, 3HDec and other HCA_3_-specific agonists, but not by GPR84-specific agonists (Figs. 8a, S11). ERK activation was detectable for 3HO but not for 3HDec (Fig. 8b). Further, in the presence of 3HO Colo680N spheroids were always less dense compared to control as determined by volume measurement of the spheroids as well as Hoechst 33342 and PI staining (Fig. 8c). This resembled our findings for HCA_3_-expressing CHO-K1 spheroids (Figs. 7a, 8c). Similarly, the observation that disruption of dyn-2 function caused less dense spheroids was also confirmed in Colo680N cells (Fig. 8d).

## Discussion

A recent study provides insight in GPR84-mediated biased agonism, with the described agonists being surrogate ligands [53]. Although several agonists are also known to activate HCA_3_, to our knowledge, no study so far aimed to understand signaling outcome of HCA_3_ activation by different agonists. The facts that GPR84 and HCA_3_ are both G_i_-coupled receptors, often co-expressed in immune cells and activated by structurally highly similar agonists raise the question: what is the evolutionary advantage of expressing two apparently redundant GPCRs in the same cell? Potential explanations could be that beyond the G_i_-protein signaling both receptors differ in downstream signal transduction, signaling kinetics and/or internalization mechanisms. Internalization and endocytosis of GPCRs are not only relevant for termination of receptor signaling but are a crucial part of the receptor-mediated signal transduction. The GTPase dyn, arrestins and several other components have been shown to play an important role in these processes [55].

Using kinetic DMR analyses, we found that dynasore, a cell-permeable non-competitive inhibitor of the GTPase activity of dyn, differentially affected signaling of both receptors when comparing their two respective agonists analyzed here [35]. Dyn is essential for clathrin-coated vesicle formation in endocytosis, for transport from the trans-Golgi network, as well as for ligand uptake through caveolae [36]. Comprehensive analyses of HCA_3_ and GPR84 downstream signaling upon activation with respective selected agonists in the presence of different inhibitors revealed the involvement of different signaling and endocytosis components (Fig. 6c).

We found that signaling and internalization of HCA_3_ upon stimulation with 3HO is dependent on dyn-2, clathrin and β-arrestin-2. In contrast, the stimulation with 3HDec, though also dependent on dyn-2, did not lead to β-arrestin-2 recruitment and did not require clathrin but rather caveolin (Fig. 6c). We observed that these differences in signal transduction influenced cell-cell adhesion as seen in 3D cell culture models (Figs. 7, 8). Further, we showed that dyn-2 is not only a crucial component in HCA_3_ signaling but also plays an important role for localization and trafficking of HCA_3_ which again influenced growth and density of spheroids (Figs. 7, 8). Interestingly, we found that, in contrast to HCA_3_, the presence of dynasore caused a sustained signaling of the evolutionarily closest HCA_3_ relatives HCA_1_ and HCA_2_ (Figure S4). Both, HCA_1_ and HCA_2_ exhibit a 22 aa and 24 aa shorter C terminus, respectively and instead of Trp^142^ (HCA_3_) an Arg at this position (HCA_1_: Arg^130^, HCA_2_: Arg^1^^42^) [25]. These structural differences are possibly responsible for the differing interaction with dyn-2.

Signaling of GPR84 induced by both agonists, C10 and 3HDec, was also dependent on dyn-2. GPR84 internalization upon activation by C10 was rather dependent on caveolin than clathrin whereas the opposite was true for 3HDec (Fig. 6c). No internalization inhibitor applied had an effect on the 3HDec-induced cAMP inhibitory signaling of GPR84 suggesting that this part of its signaling is independent of internalization (Fig. 6c). Further, barbardin, an inhibitor of the tripartite interaction between arrestin, AP2 and clathrin did not diminish cAMP inhibitory signaling or ERK activation by both agonists and no β-arrestin-2 recruitment was detectable (Figs. 4, S7D, S7E).

An interaction with dyn has also been shown for the Gβγ complex thereby modulating its activity and influencing receptor internalization as well as trafficking [50]. We applied the inhibitor gallein, which blocks Gβγ signaling without affecting GPCR-dependent Gα activation [56]. Activation of both, HCA_3_ and GPR84, by either agonist subsequently caused decreased intracellular cAMP levels through inhibition of adenylyl cyclase, which was completely lost in the presence of PTX. Nevertheless, presence of gallein still partially inhibited the 3HO and 3HDec-induced HCA_3_-mediated and the C10-induced GPR84-mediated reduction in intracellular cAMP levels (Fig. 5) suggesting an involvement of both, Gα_i_ and Gβγ, being responsible for this signaling outcome. Golgi-localized Gβγ subunits are also involved in regulating protein transport from the trans-Golgi Network to the cell surface, which is also true for dyn-2 [36, 57]. Thus, our finding that gallein also negatively affected cell surface expression of HCA_3_ indicates that, besides dyn-2, Gβγ subunits are involved in HCA_3_ trafficking (Figure S8). In contrast, gallein had no effect on the 3HDec-induced GPR84-mediated decrease in intracellular cAMP levels suggesting that activation only by C10 but not 3HDec involved Gβγ signaling that influences intracellular cAMP levels. At last, we found that GPR84-mediated ERK activation only by 3HDec persisted upon washout of agonist (Fig. 6b).

As mentioned above, GPR84 functions as an enhancer of inflammatory signaling in macrophages once inflammation is established whereas a contrary role is suggested for HCA_3_ [25, 27]. Further, both receptors are co-expressed in different types of immune cells (Table S5), and share the agonist 3HDec. Interestingly, our findings revealed marked differences in the involved signaling components depending on the activated receptor, indicating that 3HDec will induce distinct signaling events in cells co-expressing HCA_3_ and GPR84. Specifically, in contrast to GPR84, HCA_3_ signaling upon stimulation with 3HDec is internalization- as well as Gβγ-dependent, involves rac1 and ras/rho and does not persist upon agonist-washout (Fig. 6c). Signal integration of both, HCA_3_ and GPR84, could potentially play a role for immune cells to decide whether to induce a pro- or anti-inflammatory response. That this assumption could be relevant in a physiological setting is supported by our recent findings that besides 3HO and D-Phe [26] the LAB-derived metabolite D-PLA is a highly potent HCA_3_ agonist [25]. We showed that D-PLA is resorbed from gastrointestinal tract and acts as a chemoattractant for monocytes. Our results suggested that fixation of HCA_3_ provided an evolutionary advantage potentially improving the tolerance to fermented food through activation of HCA_3_ by these LAB-derived exogenous agonists while simultaneously priming the immune system to avoid infections by pathogenic bacteria [25]. Interestingly, D-PLA showed a similar activation kinetic like 3HO at HCA_3_ whereas D-Phe and L-PLA resembled the 3HDec response (Figure S9). Furthermore, 3HO originating from increased lipolysis and D-PLA originating from LAB-fermented food both activated HCA_3_ resulting in β-arrestin-2 recruitment (Figs. 4b, S10B). This differential signaling outcome upon HCA_3_ activation potentially constitutes a mechanism enabling immune cells to differentiate between endogenous (3HO), non-pathogenic compounds (D-PLA) versus compounds originating from e.g. pathogenic bacteria (3HDec from LPS or D-Phe). This is in line with the fact that pro-inflammatory signaling induced by activation of GPR84 by e.g. 3HDec will be appropriate to control infections.

Besides the different signaling outcome of HCA_3_ and GPR84 in response to the same agonist, our study also highlights differences in the signaling components and endocytic pathways induced by different agonists at the same receptor, indicating biased agonism at both HCA_3_ and GPR84. We are aware that biased agonism is dependent on cell type but basic understanding of receptor function is still deducible from signal transduction analyses in heterologous expression systems. The observed differences in endocytosis and signaling potentially result in distinct physiological responses in cells endogenously expressing HCA_3_ and/or GPR84, like e.g. cell adhesion and migration. In case of HCA_3,_ this notion is supported by the observation that 3HO but not 3HDec affected growth and density of HCA_3_-expressing spheroids. In summary, understanding the distinct effects of different agonists acting on both receptors, HCA_3_ and GPR84, poses a fundamental basis to recognize their function in immune cells.

## Conclusions

A better understanding of the components involved in signal transduction of HCA_3_, GPR84 and their downstream effectors in immune cells will be mandatory to assess their potential as drug targets. This is especially crucial since recent clinical trials have e.g. explored the potential of ligands blocking GPR84 function for the treatment of ulcerative colitis [58]. Additionally, one recent study illustrated that care has to be taken with regard to on- and off-target effects of different orthosteric and allosteric activators of GPR84 [9]. Our present study adds to these findings, highlighting differences of naturally occurring agonists not only activating GPR84 but also HCA_3_, a receptor often co-occurring with GPR84 and also found overexpressed in patients with ulcerative colitis. Future studies will have to focus on endogenously HCA_3_- and GPR84- expressing cells of the innate immune system, including neutrophils, monocytes and macrophages, to unravel how the signal of different agonists is integrated, thereby modulating pathways leading to a pro−/anti-inflammatory response, migration, phagocytosis and ROS production.

## Supplementary information

**Additional file 1: Figure S1** Basal activity and agonist-induced receptor internalization of HCA_3_ and GPR84. **Figure S2** Differential, agonist-specific dynasore-sensitivity of HCA_3_ and GPR84 in DMR analyses. **Figure S3** Concentration-response curves derived from DMR analyses of HCA_3_ and GPR84. **Figure S4** DMR analyses of HCA_1_ and HCA_2_. **Figure S5** No influence of dynasore, sucrose, gallein, MβCD, and barbardin on intracellular cAMP levels in empty vector transfected CHO-K1 cells in the presence of HCA_3_ and GPR84 agonists. **Figure S6** Time course of ERK activation for HCA_3_ and GPR84. No calcium signals detected upon stimulation of HCA_3_ and GPR84. **Figure S7** Basal activity and cAMP inhibitory signaling of HCA_3_ and GPR84 in HEK293-T cells. **Figure S8** Cell surface expression of HCA_3_, GPR84, ADBR2, and V2R in the presence of dynasore, barbardin, and gallein. **Figure S9** Dynasore-sensitivity of agonist-induced DMR responses of human, gorilla and orangutan HCA_3_. **Figure S10** Lactic acid bacteria-derived HCA_3_ agonists do not activate GPR84 and HCA_3_ recruits β-arrestin-2. **Figure S11** PTX-sensitive cAMP inhibitory response of Colo680N cells when stimulated with HCA_3_ agonists but no signal upon stimulation with GPR84 agonists. **Table S1** Primers used for GPR84, dynamin-2, HCA_3_ amplification, sequencing and introduction of epitope tags. **Table S2** Summary of E_max_ and EC_50_ values as determined from DMR, cAMP and ERK analyses of HCA_3_ and GPR84. **Table S3** Summary of cAMP data acquired for HCA_3_ and GPR84 in absence and presence of dynasore, sucrose, barbardin, MβCD and gallein. **Table S4.** Summary of ERK data acquired for HCA_3_ and GPR84 in absence and presence of dynasore, sucrose, barbardin, MβCD, gallein, ZA, NSC23766 and Ly294002. **Table S5.** TPM values as downloaded from Expression Atlas: https://www.ebi.ac.uk/gxa/home . **Supplementary Results and Discussion. (PDF 5177 kb)**

## Data Availability

All data generated or analyzed during this study are included in this published article and its supplementary information files.

## Abbreviations

3HDec: 3-hydroxydecanoic acid
3HO: 3-hydroxyoctanoic acid
ADRB2: β_2_-adrenergic receptor
AP2: Adaptor protein complex 2;
C10: Decanoic acid
cAMP: Cyclic adenosine monophosphate
DMR: Dynamic mass redistribution
D-PLA: D-phenyllactic acid
dyn: Dynamin
ERK: Extracellular signal–regulated kinases
fsk: Forskolin
GPCR: G protein-coupled receptor
HCAR: Hydroxycarboxylic acid receptor
ILA: Indole 3-lactic acid
IPBT5CA: 1-isopropylbenzotriazole-5-carboxylic acid
LAB: Lactic acid bacteria
MCFA: Medium-chain fatty acid
PI: Propidium iodide
PI3K: PI3-kinase
PTX: Pertussis toxin
V2R: V2 vasopressin receptor
wt: Wildtype
ZA: Zoledronic acid
