# Supplementary material for: Natural biased signaling of hydroxycarboxylic acid receptor 3 and G protein-coupled receptor 84
Source: Cell Commun Signal. 2020 Feb 26;18:31. doi: 10.1186/s12964-020-0516-2 (PMC7045412; doi:10.1186/s12964-020-0516-2)
Supplement: Supplementary file 2 — Additional file 1: Figure S1 Basal activity and agonist-induced receptor internalization of HCA3 and GPR84. Figure S2 Differential, agonist-specific dynasore-sensitivity of HCA3 and GPR84 in DMR analyses. Figure S3 Concentration-response curves derived from DMR analyses of HCA3 and GPR84. Figure S4 DMR analyses of HCA1 and HCA2. Figure S5 No influence of dynasore, sucrose, gallein, MβCD, and barbardin on intracellular cAMP levels in empty vector transfected CHO-K1 cells in the presence of HCA3 and GPR84 agonists. Figure S6 Time course of ERK activation for HCA3 and GPR84. No calcium signals detected upon stimulation of HCA3 and GPR84. Figure S7 Basal activity and cAMP inhibitory signaling of HCA3 and GPR84 in HEK293-T cells. Figure S8 Cell surface expression of HCA3, GPR84, ADBR2, and V2R in the presence of dynasore, barbardin, and gallein. Figure S9 Dynasore-sensitivity of agonist-induced DMR responses of human, gorilla and orangutan HCA3. Figure S10 Lactic acid bacteria-derived HCA3 agonists do not activate GPR84 and HCA3 recruits β-arrestin-2. Figure S11 PTX-sensitive cAMP inhibitory response of Colo680N cells when stimulated with HCA3 agonists but no signal upon stimulation with GPR84 agonists. Table S1 Primers used for GPR84, dynamin-2, HCA3 amplification, sequencing and introduction of epitope tags. Table S2 Summary of Emax and EC50 values as determined from DMR, cAMP and ERK analyses of HCA3 and GPR84. Table S3 Summary of cAMP data acquired for HCA3 and GPR84 in absence and presence of dynasore, sucrose, barbardin, MβCD and gallein. Table S4. Summary of ERK data acquired for HCA3 and GPR84 in absence and presence of dynasore, sucrose, barbardin, MβCD, gallein, ZA, NSC23766 and Ly294002. Table S5. TPM values as downloaded from Expression Atlas: https://www.ebi.ac.uk/gxa/home . Supplementary Results and Discussion. (PDF 5177 kb) [file 12964_2020_516_MOESM1_ESM.pdf]

Supplementary Material

Natural biased signaling of hydroxycarboxylic acid receptor 3 and G protein-coupled receptor 84

Anna Peters[1], Philipp Rabe[1], Petra Krumbholz[1], Hermann Kalwa[2], Robert Kraft[3], Torsten Schöneberg[1], Claudia Stäubert[1]*

[1] Rudolf Schönheimer Institute of Biochemistry, Medical Faculty, Leipzig University, Johannisallee 30, 04103 Leipzig, Germany

[2] Rudolf Böhm Institute of Pharmacology and Toxicology, Medical Faculty, Leipzig University, Härtelstraße 16-18, 04107 Leipzig, Germany

[3] Carl Ludwig Institute for Physiology, Medical Faculty, Leipzig University, 04103 Leipzig, Germany

Running Title: HCA_3 and GPR84 biased signaling

The authors have declared that no competing interests exist.

Figure S1

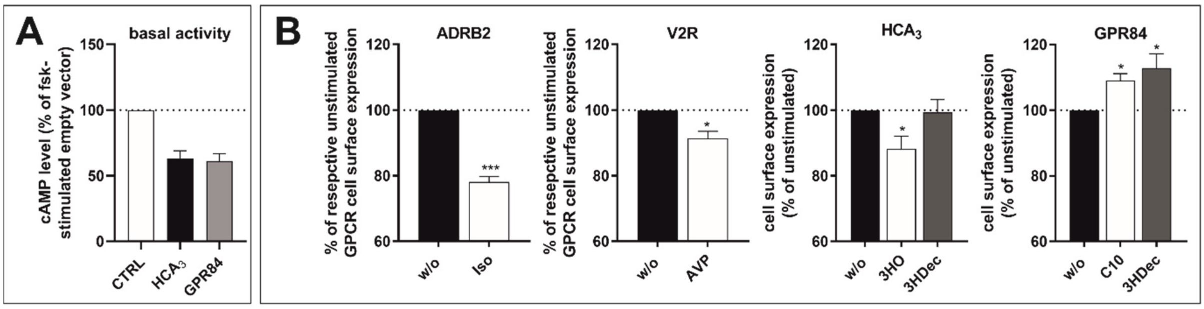

Figure S1 Basal activity and agonist-induced receptor internalization of HCA$_3$ and GPR84. CHO-K1 cells were transiently transfected with receptor constructs or empty vector (control). (A) Both, HCA$_3$ and GPR84, exhibited a basal cAMP inhibitory activity (cAMP level of empty vector-transfected forskolin-stimulated cells is set to 100 %) (B) The β$_2$-adrenergic receptor (ADBR2) and V2 vasopressin receptor (V2R) served as control, which showed 22 % and 9 % reduction of cell surface expression upon stimulation with 10 µM isoprenalin and 10 µM arginine vasopressin (AVP), respectively. 100 µM 3HO but not 100 µM 3HDec induced a significant reduction in cell surface expression of HCA$_3$. GPR84 cell surface expression levels were increased in presence of 100 µM C10 and 100 µM 3HDec. Cell surface expression levels of respective receptor construct in absence of agonist is set to 100 %. Data is shown as mean ± SEM of at least three independent experiments each carried out in triplicates. * P ≤ 0.05; ** P ≤ 0.01.

Figure S2

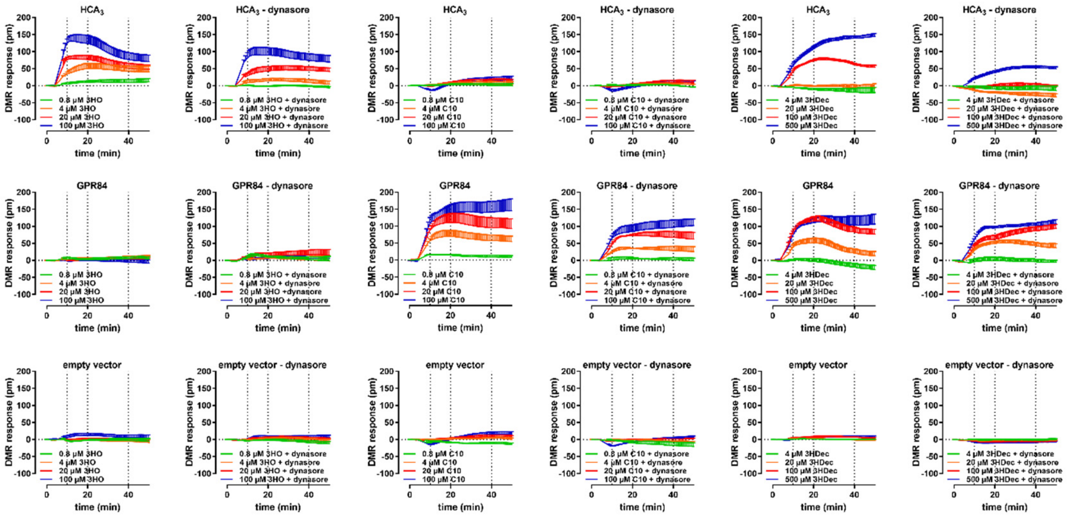

Figure S2 Differential, agonist-specific dynasore-sensitivity of HCA$_3$ and GPR84 in DMR analyses. CHO-K1 cells were transiently transfected with receptor constructs or empty vector, seeded in fibronectin-coated Epic plates and DMR responses were recorded. 3-hydroxyoctanoic acid (3HO) activated HCA$_3$ but not GPR84. Decanoic acid (C10) specifically activated GPR84 but not HCA$_3$. 3-hydroxydecanoic acid (3HDec) activated both, HCA$_3$ and GPR84. No DMR response upon agonist stimulation was observed in cells transfected with empty vector. 80 µM dynasore was used. Shown is the agonist-induced wavelength shift in pm as mean ± SEM of three to five independent experiments, each carried out in triplicates. DMR at time points 10 min, 20 min and 40 min were extracted to generate Figure 1B and Figure S3.

Figure S3

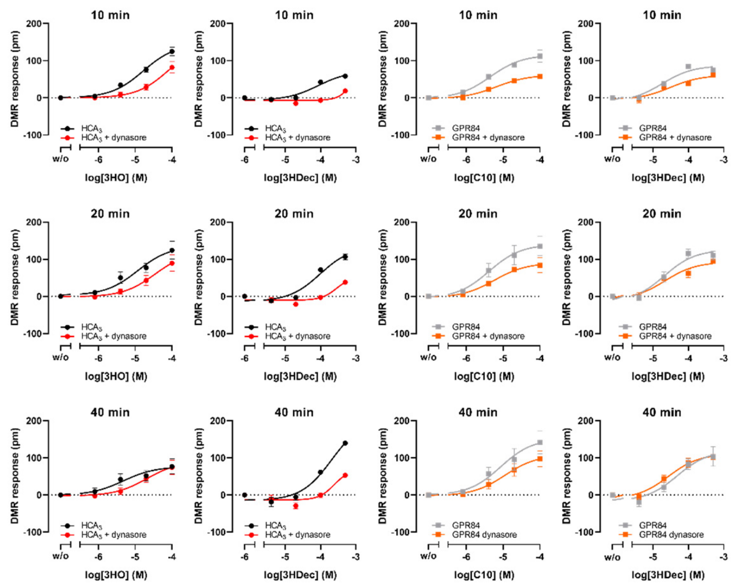

Figure S3 Concentration-response curves derived from DMR analyses of HCA_3 and GPR84. CHO-K1 cells were transiently transfected with receptor constructs or empty vector, seeded in fibronectin-coated Epic plates and DMR responses were recorded. Concentration-response curves in absence and presence of dynasore were derived from time points 10 min, 20 min and 40 min (Figure S2). Shown is the agonist-induced wavelength shift in pm as mean ± SEM of three to five independent experiments, each carried out in triplicates.

Figure S4

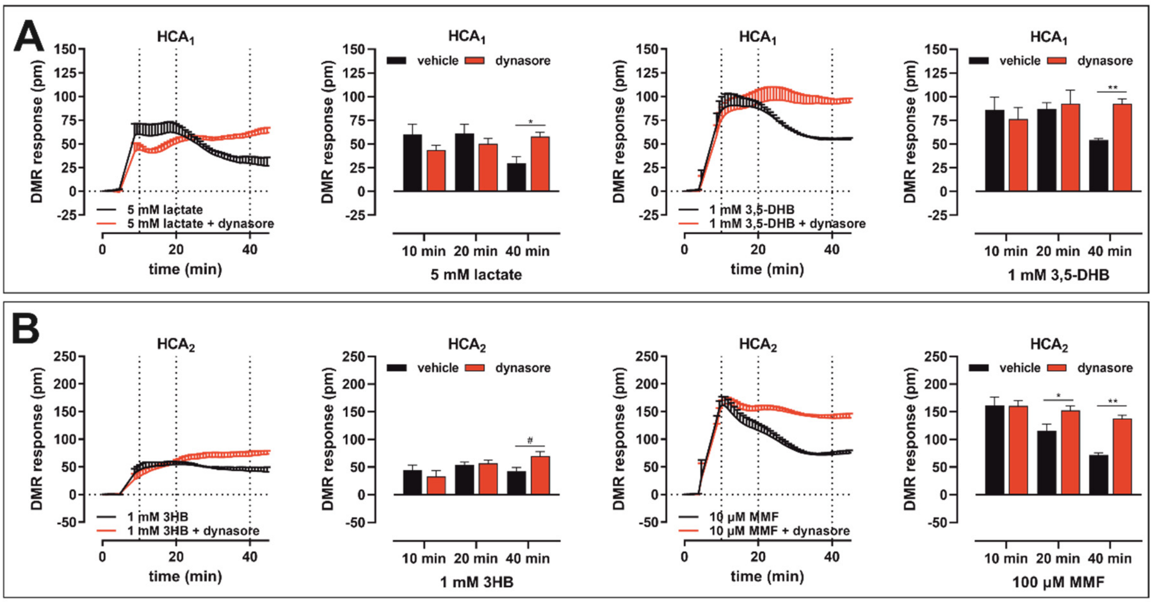

Figure S4 DMR analyses of HCA_1 and HCA_2. CHO-K1 cells were transiently transfected with receptor constructs or empty vector, seeded in fibronectin-coated Epic plates and DMR responses were recorded. (A) The lactate- and 3,5-dihydroxybenzoic acid (3,5-DHB)-induced DMR response of HCA_1 transfected CHO-K1 cells and (B) the 3-hydroxybutyrate (3HB) and monomethylfumarate induced response of HCA_2 transfected CHO-K1 cells were at later time points affected by presence of dynasore. Shown is the agonist-induced wavelength shift in pm as mean ± SEM of three to five independent experiments, each carried out in triplicates.

Figure S5

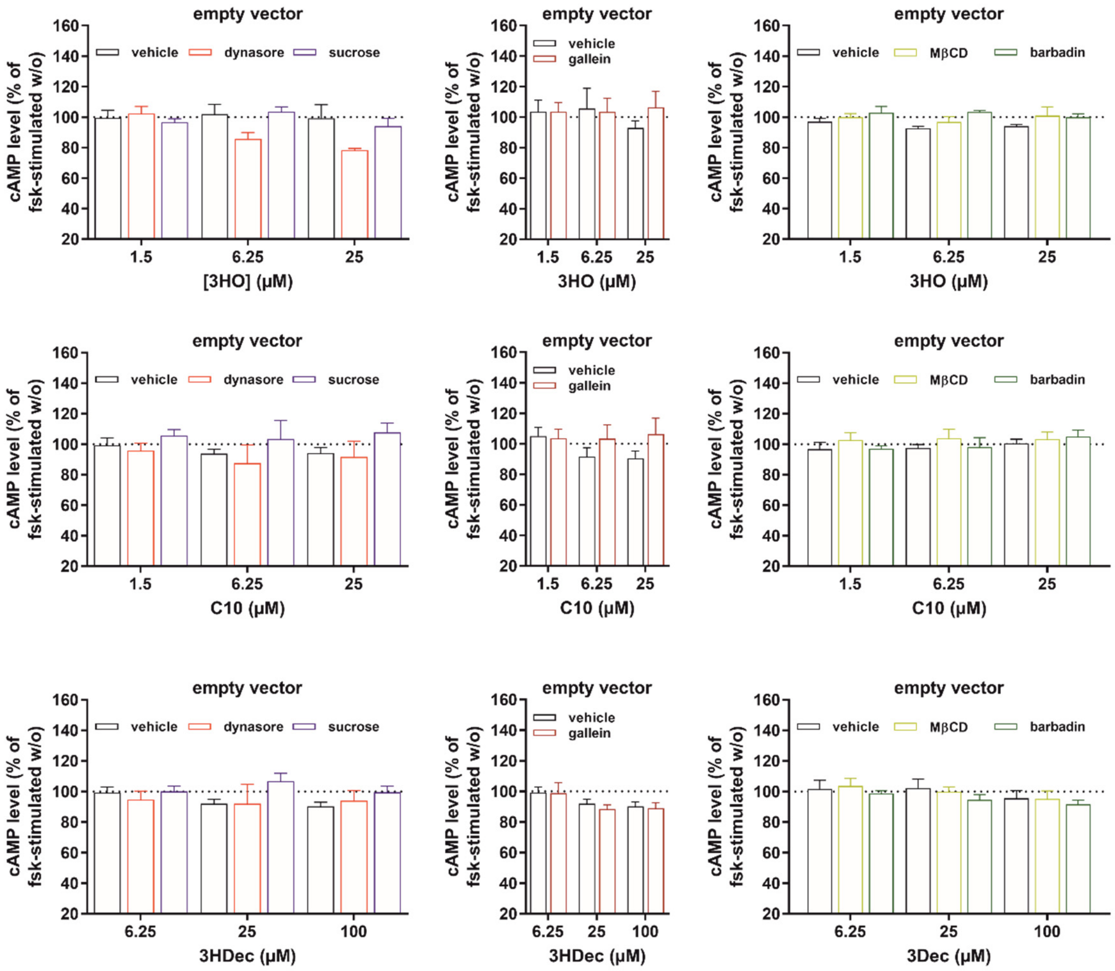

Figure S5 No influence of dynasore, sucrose, gallein, MβCD, and barbardin on intracellular cAMP levels in empty vector transfected CHO-K1 cells in the presence of HCA_3 and GPR84 agonists. Fsk-induced cAMP level of empty vector-transfected cells in absence of agonist is set 100 %.

Figure S6

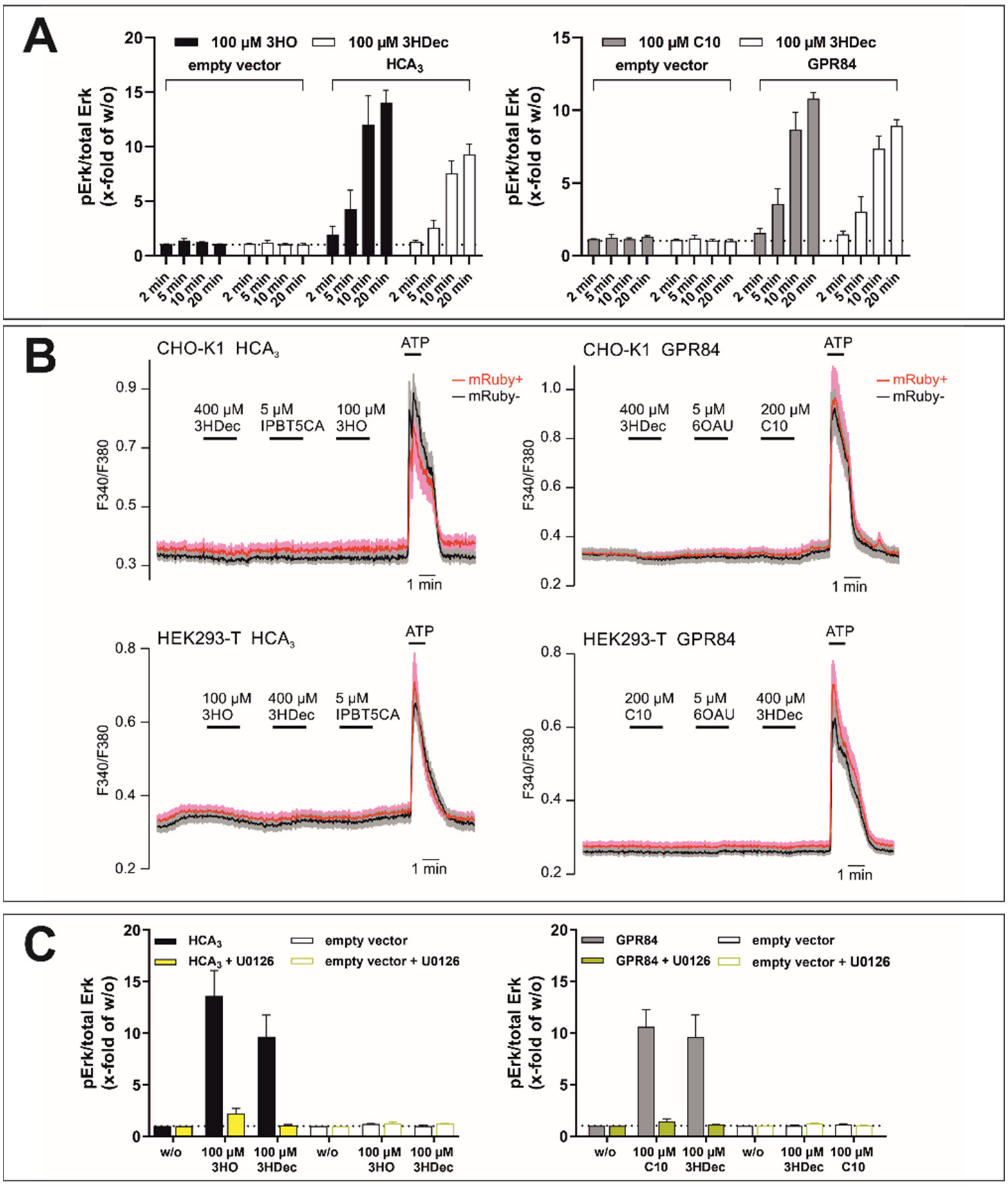

Figure S6 Time course of ERK activation for HCA$_3$ and GPR84. No calcium signals detected upon stimulation of HCA$_3$ and GPR84. (A) Agonist-induced phosphorylation of endogenous ERK 1/2 in cellular lysates of HCA$_3$ or GPR84 transfected CHO-K1 cells over time. (B) Fura 2-based calcium imaging experiments were performed in CHO-K1 and HEK293-T cells transiently transfected with either GPR84 or HCA$_3$ (both mRuby-tagged). The fluorescence ratio (F340/F380) represents the time course of calcium in mRuby-positive (mRuby+) and mRuby-negative (mRuby-) cells from the same coverslip. Each trace represents the average calcium signal ± SEM from 10 CHO-K1 or 20 HEK293-T cells. The GPR84 agonists 3HDec (400 µM), 6OAU (5 µM), and C10 (200 µM) were unable to induce calcium signals. The endogenous agonist ATP (100 µM) evoked prominent calcium responses. Similarly, the HCA$_3$ agonists 3HDec (400 µM), IPBT5CA (5 µM), and 3HO (100 µM) were ineffective, whereas ATP induced strong signals. (C) ERK activation of both HCA$_3$ and GPR84 was completely inhibited by 10 µM U0126. (A, C) Data is given as mean ± SEM of at least three independent experiments each carried out in triplicates.

Figure S7

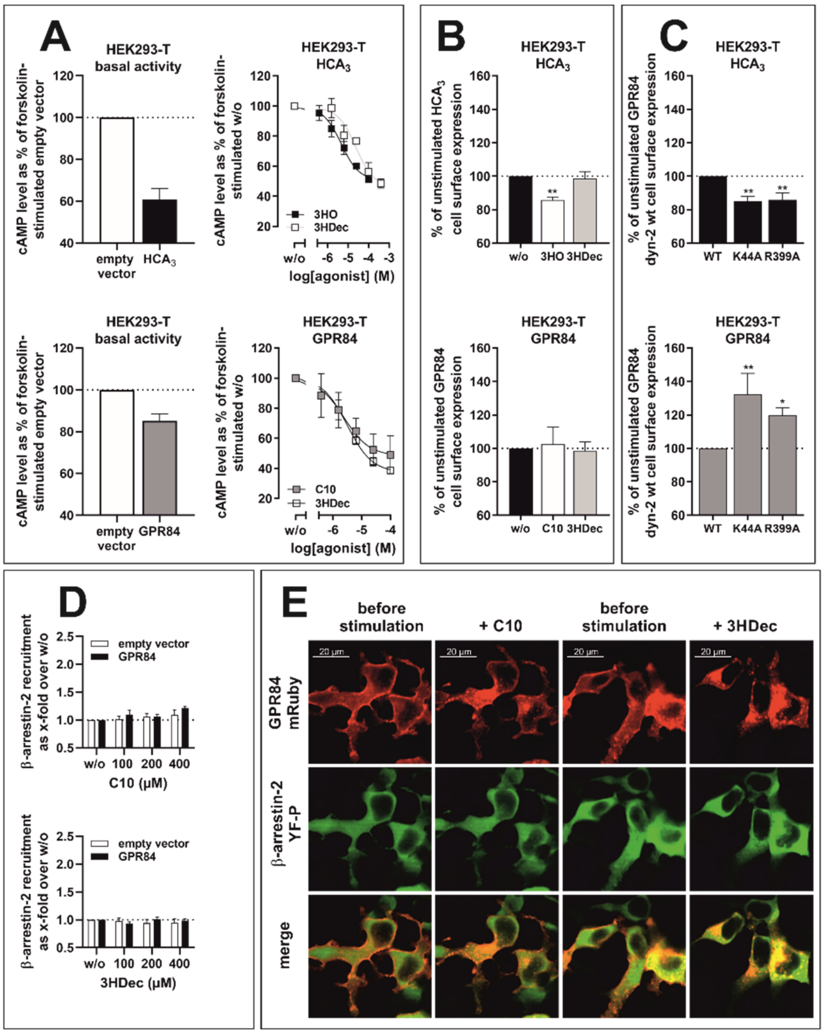

Figure S7 Basal activity and cAMP inhibitory signaling of HCA_3 and GPR84 in HEK293-T cells. (A) HCA_3 and GPR84 showed a basal activity in HEK293-T cells (cAMP level of empty vector-transfected fsk-stimulated cells is set to 100 %). Both receptors were activated in a concentration-dependent manner by their respective agonists as determined by cAMP inhibition assays (cAMP level of HCA_3 and GPR84 transfected cells in absence of agonist is set to 100 %, respectively.) (B) 100 µM 3HO but not 100 µM 3HDec induced a significant reduction in cell surface expression of HCA_3. No reduction of cell surface expression was measured upon stimulation of GPR84 with 100 µM 3HDec and 100 µM C10. Cell surface expression level in absence of agonist is set to 100 %. (C) In comparison to dyn-2 wt, cell surface expression of HCA_3 but not GPR84 was significantly reduced when K44A or R399A were co-transfected. Cell surface expression level of dyn-2 wt co-transfected cells is set to 100 %. (D) HEK293-T cells stably expressing β-arrestin-2-EA cells transiently transfected with GPR84 were stimulated with C10 and 3HDec. Quantification of β-arrestin-2 recruitment using the PathHunter β-arrestin assay (Eurofins DiscoverX) showed no recruitment of β-arrestin-2 by GPR84 following agonist stimulation. Luminescence of GPR84 or empty vector transfected cells in absence of agonist is set 1, respectively. (E) Live-cell images of HEK293-T cells co-expressing GPR84-mRuby (red) and β-arrestin-2-YFP (green) were acquired before stimulation and 30 min post-stimulation with 100 µM C10 or 100 µM 3HDec. (A-D) Data is given as mean ± SEM of at least three independent experiments each carried out in triplicates. ** $P \leq 0.01$.

Figure S8

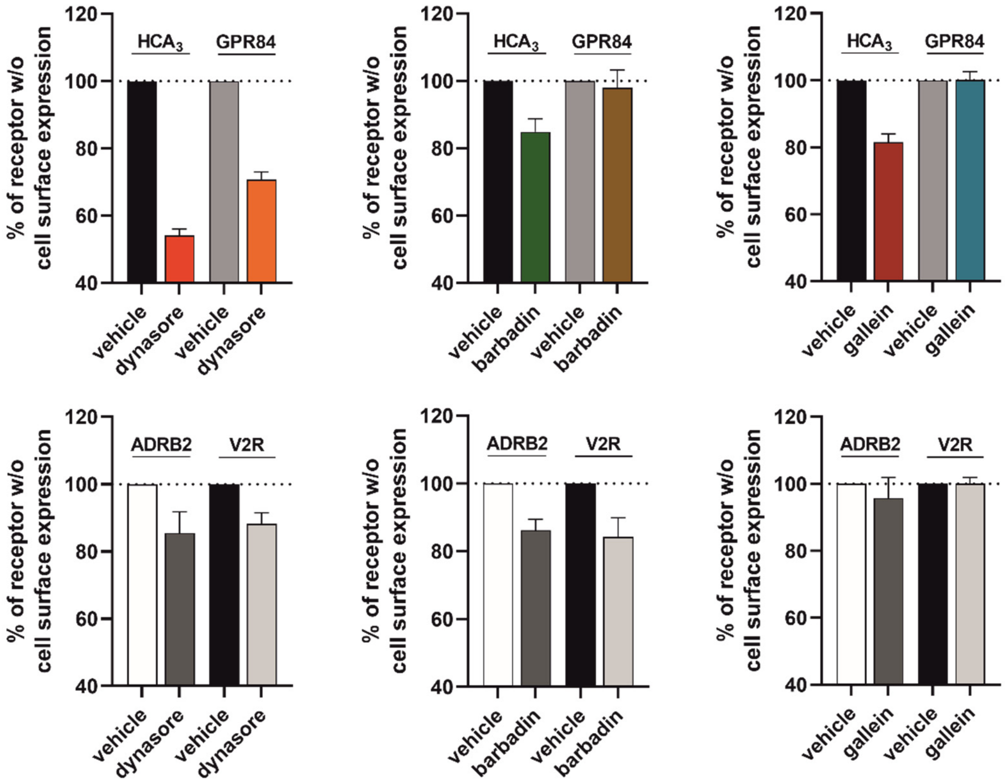

Figure S8 Cell surface expression of HCA₃, GPR84, ADBR2, and V2R in the presence of dynasore, barbardin, and gallein. CHO-K1 cells were transiently transfected with receptor constructs and cell surface expression in absence of agonists but presence of 80 µM dynasore, 50 µM gallein and 100 µM barbardin was determined. Cell surface expression level of respective receptor construct in absence of inhibitor is set to 100 %. Data is shown as mean ± SEM of four independent experiments each carried out in triplicates.

Figure S9

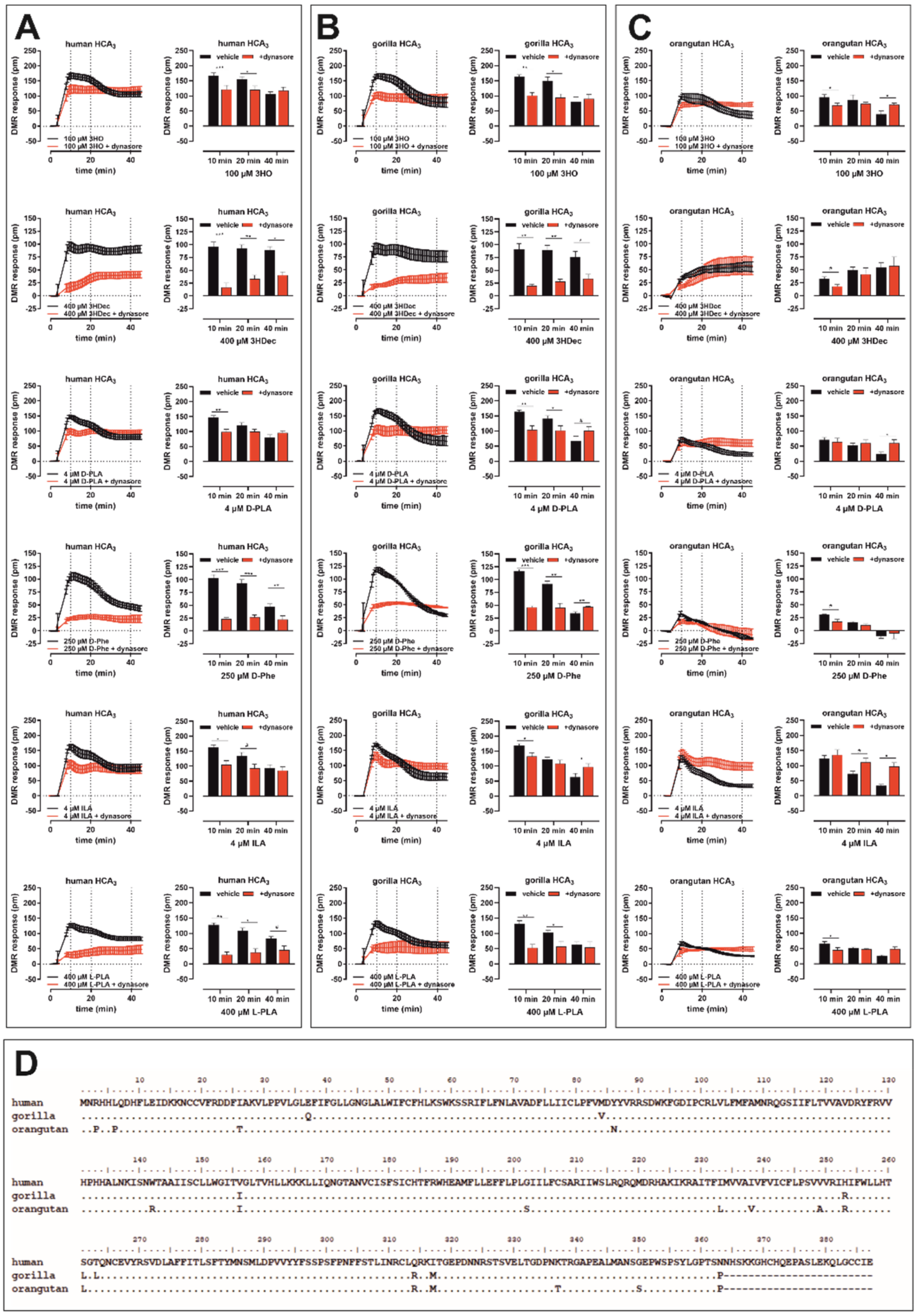

Figure S9 Dynasore-sensitivity of agonist-induced DMR responses of human, gorilla and orangutan HCA_3. CHO-K1 cells were transiently transfected with HCA_3 orthologs, seeded in fibronectin-coated Epic® plates and DMR responses were recorded. Presence of dynasore similarly affected the DMR response of human HCA_3 (A) and gorilla HCA_3 (B) expressing CHO-K1 cells to 3HO, D-phenyllactic acid (D-PLA) and indole 3-lactic acid (ILA). In contrast, responses to 3HDec and D-phenylalanine (D-Phe) and L-phenyllactic acid (L-PLA) were almost completely diminished in presence of dynasore. (C) Orangutan HCA_3 DMR responses to different agonists in presence of dynasore showed a distinct pattern when compared to human and gorilla HCA_3. A rather prolonged activation of the receptor in presence of dynasore was observed as also seen for human HCA_1 and HCA_2 (Figures S4A, S4B). Shown is the agonist-induced wavelength shift in pm as mean ± SEM of three independent experiments, each carried out in triplicates. (D) Amino acid sequence alignment of human, gorilla and orangutan HCA_3.

Figure S10

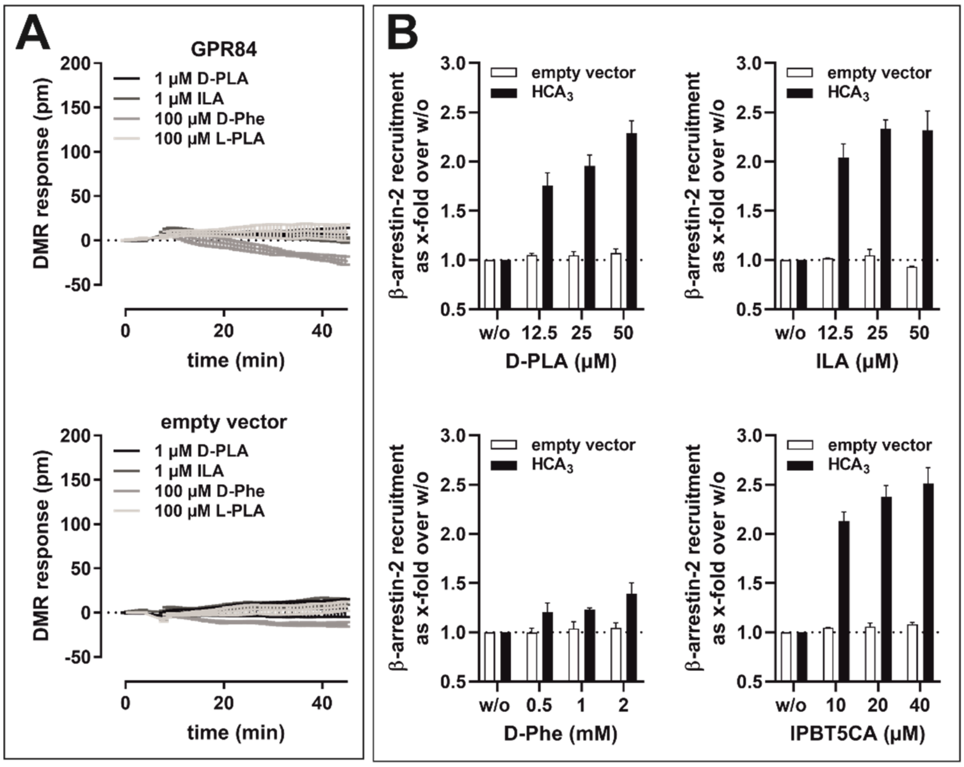

Figure S10 Lactic acid bacteria-derived HCA_3 agonists do not activate GPR84 and HCA_3 recruits β-arrestin-2 (A) CHO-K1 cells were transiently transfected with receptor constructs or empty vector, seeded in fibronectin-coated Epic plates and DMR responses were recorded. None of the depicted HCA_3 agonists: D-phenyllactic acid (D-PLA), indole 3-lactic acid (ILA) and D-phenylalanine (D-Phe) activated GPR84 or empty vector-transfected CHO-K1 cells. Shown is the agonist-induced shift in pm as mean ± SEM of three to five independent experiments, each carried out in triplicates. (B) HEK293-T cells stably expressing β-arrestin-2-EA were transiently transfected with HCA_3. Quantification of β-arrestin-2 recruitment using the PathHunter β-arrestin assay (Eurofins DiscoverX) shows recruitment of β-arrestin-2 by HCA_3 following D-PLA, ILA, IPBT5CA but not D-Phe stimulation. Luminescence of HCA_3 or empty vector-transfected cells in absence of agonist is set 1, respectively. Data is given as mean ± SEM of three independent experiments each carried out in triplicates.

Figure S11

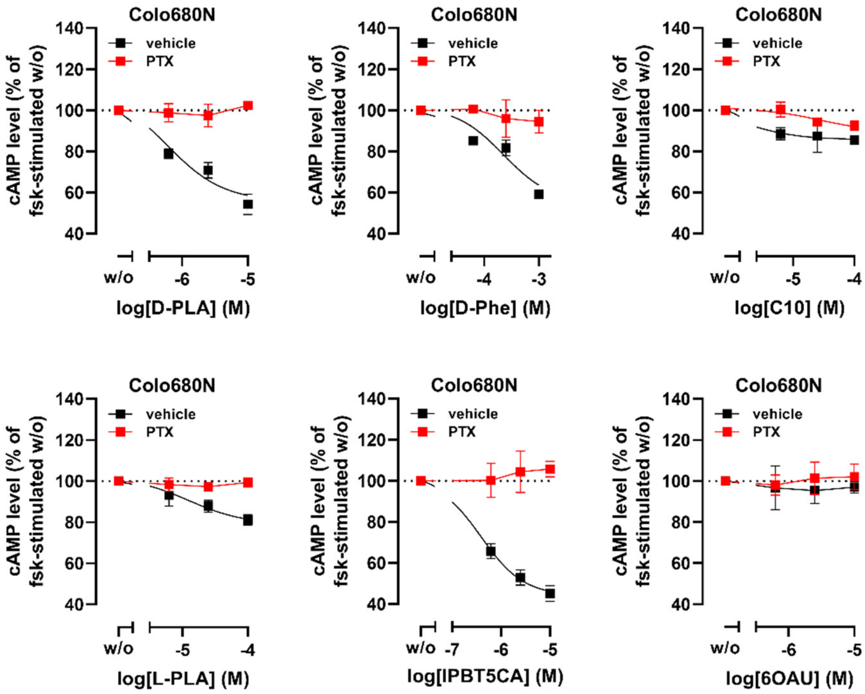

Figure S11 PTX-sensitive cAMP inhibitory response of Colo680N cells when stimulated with HCA$_3$ agonists but no signal upon stimulation with GPR84 agonists. cAMP inhibition assays in the absence and presence of 100 ng/ ml pertussis toxin (PTX) confirmed presence of HCA$_3$ and absence of GPR84 in Colo680N cells. Data is shown as mean ± SEM of three independent experiments each carried out in triplicates. IPBT5CA, 1-(1-methylethyl)-1H-benzotriazole-5-carboxylic acid; 6OAU, 6-(octylamino)-2,4(1H,3H)-pyrimidinedione

Supplementary Table S1

Primers used for GPR84, dynamin-2, HCA_3 amplification, sequencing and introduction of epitope tags

ID	sequence (5´- 3´)	purpose
789	GTGCAAATCAAAGAACTGCTCCTC	pcDps forward (amplification/sequencing)
790	CCTGGTTCTTTCCGCCTCAGAAG	pcDps reverse (amplification/sequencing)
2291	CGCCGCACTAGTTCACTTATCGTCATCGTCCTTATAGTC	FLAG-uni-*Spe I*_AS
2292	CGCCGGGTACCTCACTCACTTATCGTCATCGTCCTTATAGTC	FLAg-uni-*KpnI*_AS
2285	CGCGAATTCCCCACCATGTACCCCTACGACGTCCCCGACTACGCC	HA-uni-Kozak-*Eco RI*_S
2289	CGCCCCGGGCCCACCATGTACCCCTACGACGTCCCCGACTACGCC	HA-uni-Kozak-*Xma I*_S
311	TCCACCCTCTGCCTCTTTAG	human GPR84-33-5UTR-S
312	TGTTGTGAAAATGCCCACAT	human GPR84-105-3UTR-AS
313	GATAGTGCTGGCACTGGTGA	human GPR84-408-ORF-S
314	GCTCAGATGAAATCCCCTCA	human-GPR84-772-ORF-AS
315	TGAAGCCTAACTGTCCACCA	human GPR84-6-mRNA-S
316	GCTGGGGAGAGATTGTTGTG	human GPR84-1411-mNA-AS
317	ACGTCCCCGACTACGCCTGGAACAGCTCTGACGCC	human GPR84- HA-adaptor
318	TCACTTATCGTCATCGTCCTTATAGTCATGGAGCCTATGGAAACTCCG	human GPR84-FLAG-adaptor
596	TGAGGATGGCTGTCTCG	mRuby-342-Seq-S
597	GGCATCTTGATGTTCCCG	mRuby-581-Seq-AS
586	GCCAGAGCGCCGGCAAGAGTTCGGTGCTC	rat_dynamin-2_Lys44-S
587	GAGCACCGAACTCTTGCCGGCGCTCTGGC	rat_dynamin-2_Lys44-AS
590	GAACATCCACGGAGTCGCGACGGGGCTCTTCAC	rat_dynamin-2_Ala399_S
591	GTGAAGAGCCCCGTCGCGACTCCGTGGATGTTC	rat_dynamin-2_Ala399_AS
604	AACCGAATTCCCCACCATGGGCAACCGCG	rat_dynamin-2_EcoRI-Kozak-S
605	CAGTACTAGTTCAGTCGAGCAGGGACGG	rat_dynamin-2_TGA-SpeI-AS
606	CGTCCCTGCTCGACGTGAGCAAGGGCGA	rat_dynamin-2_YFP-S
607	TCGCCCTTGCTCACGTCGAGCAGGGACG	rat_dynamin-2_YFP-AS
608	CAGTACTAGTTTACTTGTACAGCTCGTCCA	YFP-SpeI_TGA-AS
609	ACTTGGTGGACTCCTATGTG	rat_dynamin-2-S-1989S-seq
610	GGACCACAGAATGTGTTTGC	rat_dynamin-2-S-2456S-seq
576	AACCGAATTCCCCACCATGAATCGGCACCATCTG	EcoRI-ATG-HCAR3-sense
711	ATTGCTAGCCCCACCATGAATCGGC	HCAR3-PK1-NheI-S
712	ACCTCCCAATTCGAAGCTTGACTCGATGCAACA	HCAR3-PK1-HindIII-AS
719	ATTGCTAGCCCCACCATGTGGAACAGC	GPR84-PK1-NheI-S
720	ACCTCCCAATTCGAGATCTGAATGGAGCCTATG	GPR84-PK1-BglII-AS
577	CTCTTCGCCCTTAGACACCTCGATGCAACAGCCC	HCAR3-mRuby-AS
578	GGGCTGTTGCATCGAGGTGTCTAAGGGCGAAGAG	HCAR3-mRuby-S

Supplementary Table S2

CHO-K1 cells were transfected with receptor constructs and agonist-induced DMR response, inhibition of cAMP accumulation and ERK activation was determined (see Methods). E_{max} and EC_{50} values were determined from concentration-response curves of agonists 3HO, 3HDec and C10 using GraphPad Prism. Data are given as mean with 95% confidence interval of n ≥ 3 independent experiments each performed in triplicates.

	**3HO**		**3HDec**		**C10**	
	E_{max} (95% CI)	EC_{50} (µM) (95% CI)	E_{max} (95% CI)	EC_{50} (µM) (95% CI)	E_{max} (95% CI)	EC_{50} (µM) (95% CI)
**HCA_3**						
**EPIC**	***DMR shift in pm***		***DMR shift in pm***		***DMR shift in pm***	
10 min	144.6 (119.7 – 183.5)	16.5 (7.0 – 41.7)	71.9 (58.1 – 90.4)	84.4 (43.3 – 175.8)	n.d.	n.d.
20 min	124.2 (62.6 – 185.9)	8.0 (2.3 – 27.2)	133.7 (108.4 – 168.9)	96.7 (51.0 – 196.6)	n.d.	n.d.
40 min	77.0 (24.4 – 129.5)	11.6 (1.78 – 70.7)	139.5 (113.1 – 165.8)	89.6 (53.5 – 152.3)	n.d.	n.d.
**cAMP**	***% of w/o agonist***		***% of w/o agonist***		***% of w/o agonist***	
	22.1 (19.4 – 24.8)	1.6 (0.9 – 3.1)	30.1 (21.5. – 39.9)	28.8 (17.8 – 46.6)	n.d.	n.d.
**ERK**	***x-fold of w/o***		***x-fold of w/o***		***x-fold of w/o***	
	7.0 (6.2 – 7.8)	8.7 (1.7 – 19.2)	6.8 (6.0 – 7.6)	54 (25.3 – 81.7)	n.d.	n.d.
**GPR84**						
**EPIC**	***DMR shift in pm***		***DMR shift in pm***		***DMR shift in pm***	
10 min	n.d.	n.d.	86.8 (74.5 – 100.3)	18.1 (8.9 - 35.9)	115.6 (94.1 – 144.4)	4.5 (1.2 – 19.8)
20 min	n.d.	n.d.	125.3 (102.3 – 151.7)	20.7 (8.3 – 50.5)	140.1 (103.5 – 198.4)	4.1 (0.66 – 37.8)
40 min	n.d.	n.d.	119.4 (82.3 – 177.3)	48.0 (13.0 – 205.8)	142.1 (61.8 – 222.5)	6.7 (1.4 – 34.0)
**cAMP**	***% of w/o agonist***		***% of w/o agonist***		***% of w/o agonist***	
	n.d.	n.d.	30.5 (24.4 – 36.7)	9.8 (5.8 – 16.6)	30.2 (25 – 35.4)	3.4 (2.4 – 4.8)
**ERK**	***x-fold of w/o***		***x-fold of w/o***		***x-fold of w/o***	
	n.d.	n.d.	2.8 (2.5 – 3.1)	32.2 (12.4 - 47.5)	3.0 (2.7 – 3.3)	7.9 (3.8 – 11.8)

Supplementary Table S3

CHO-K1 cells were transfected with receptor constructs and agonist-induced inhibition of cAMP accumulation was determined (see Methods). Data are given as cAMP level (% of forskolin stimulated w/o agonist) for each receptor construct (mean ± SEM of n ≥ 3 independent experiments each performed in triplicates).

**cAMP**	vehicle	80 µM dynasore	0.4 M sucrose	vehicle	100 µM barbardin	3 mM MβCD	vehicle	50 µM gallein
**HCA3**								
1.5 µM 3HO	61.5 ± 6.1	91.7 ± 2.9	72.1 ± 6.2	82.8 ± 1.7	92.4 ±3.4	91.8 ± 2.0	58.2 ± 2.1	77.8 ± 4.8
6.25 µM 3HO	34.7 ± 2.1	54.9 ± 7.9	45.3 ± 5.6	66.7 ± 0.6	77.2 ± 3.4	78.3 ± 2.1	47.6 ± 1.9	61.1 ± 1.6
25 µM 3HO	27.5 ± 3.9	48.9 ± 4.2	33.2 ± 3.1	57.1 ± 3.4	68.2 ± 4.1	67.0 ± 2.4	38.6 ± 1.5	44.3 ± 0.7
6.25 µM 3HDec	77.9 ± 4.5	106.7 ± 8.1	92.1 ± 1.4	86.8 ± 2.8	88.0 ± 4.3	100.9 ± 2.4	75.2 ± 3.0	100.4 ± 7.2
25 µM 3HDec	52.6 ± 4.4	105.4 ± 8.5	74.3 ± 2.7	82.7 ± 1.7	81.9 ± 2.5	91.4 ±2.1	65.9 ± 5.0	89.1 ± 5.0
100 µM 3HDec	53.1 ± 5.5	77.9 ± 4.0	55.3 ± 5.5	73.0 ± 4.0	68.4 ± 4.6	84.5 ±2.1	53.7 ± 2.9	66.1 ± 4.6
**GPR84**								
1.5 µM C10	76.7 ± 4.0	92.8 ± 7.3	77.4 ± 1.8	74.8 ± 3.6	80.5 ± 4.8	90.4 ±3.0	72.6 ± 3.4	97.1 ± 5.1
6.25 µM C10	47.4 ± 1.3	61.8 ± 4.2	50.4 ± 2.6	61.1 ± 2.8	66.8 ± 3.3	78.7 ± 1.6	64.2 ± 1.9	85.5 ± 2.9
25 µM C10	33.4 ± 0.3	46.5 ± 3.7	35.0 ± 1.0	47.0 ±1.2	55.3 ± 3.5	64.5 ± 3.2	51.5 ±3.0	67.9 ± 4.8
6.25 µM 3HDec	67.5 ± 5.0	75.9 ± 1.6	78.5 ± 7.1	47.1 ± 3.9	46.2 ±2.2	48.4 ± 2.6	56.0 ± 2.4	56.4 ± 3.2
25 µM 3HDec	49.2 ± 4.9	52.0 ± 2.7	47.4 ± 3.1	36.0 ± 4.0	33.9 ±2.2	36.0 ± 3.0	44.0 ±2.5	45.6 ± 2.2
100 µM 3HDec	36.8 ± 5.6	33.3 ± 4.3	34.4 ± 3.3	30.8 ± 1.9	27.9 ±1.1	27.5 ±2.0	47.2 ±2.3	42.1 ±2.4
**empty vector**								
1.5 µM 3HO	99.5 ± 5.0	102.4 ± 4.5	96.4 ± 2.6	97.0 ± 2.1	102.9 ± 4.1	100.0 ± 2.4	103.6 ±7.5	103.6 ± 6.0
6.25 µM 3HO	101.9 ± 6.5	85.6 ± 4.3	103.6 ± 3.1	92.6 ± 1.4	103.3 ± 1.1	96.9 ± 3.5	105.6 ± 13.3	103.4 ± 9.0
25 µM 3HO	99.2 ± 8.9	78.3 ± 1.2	94.0 ± 5.1	94.0 ± 1.2	100.0 ± 2.2	100.9 ± 5.9	92.9 ± 4.7	106.4 ± 10.5
1.5 µM C10	99.2 ± 4.9	95.6 ± 5.1	105.7 ± 3.8	96.7 ± 4.4	97.1 ± 1.9	102.8 ± 4.7	105.1 ± 5.8	103.6 ± 6.0
6.25 µM C10	93.8 ± 2.9	87.5 ± 11.9	103.4 ± 12.1	97.4 ±2.3	98.0 ± 6.3	104.0 ± 5.8	91.6 ± 5.8	103.4 ± 9.0
25 µM C10	94.1 ± 3.7	91.6 ± 10.3	107.7 ± 6.2	100.6 ± 2.6	105.1 ± 4.1	103.2 ± 4.7	90.5 ± 4.7	106.4 ± 10.5
6.25 µM 3HDec	99.3 ± 3.5	94.7 ± 5.5	100.1 ± 3.4	101.7 ± 5.6	98.7 ± 1.7	103.6 ± 4.8	99.3 ± 3.5	98.9 ± 6.8
25 µM 3HDec	92.0 ± 2.9	92.1 ± 12.6	106.7 ± 5.2	102.3 ±5.9	94.5 ± 3.5	99.9 ± 3.2	92.0 ± 2.9	88.5 ± 2.7
100 µM 3HDec	90.2 ± 2.8	93.9 ± 6.6	99.5 ± 4.0	95.5 ± 5.0	91.6 ± 2.6	95.0 ± 5.3	90.2 ± 2.8	89.1 ± 3.5

Supplementary Table S4

CHO-K1 cells were transfected with receptor constructs and ERK activation was determined (see Methods). Data are given as pERK/total ERK (x-fold of w/o agonist) for each receptor construct (mean ± SEM of n ≥ 3 independent experiments each performed in triplicates).

**ERK**	vehicle	80 µM dynasore	0.4 M sucrose	vehicle	100 µM barbardin	3 mM MβCD	vehicle	50 µM gallein	vehicle	25 µM ZA	vehicle	100 µM NSC23766	25 µM Ly294002
**HCA_3**													
25 µM 3HO	n.d.	n.d.	n.d.	2.4 ± 0.2	1.9 ± 0.2	2.6 ± 0.1	3.0 ± 0.1	1.4 ± 0.1	2.4 ±0.2	2.0 ± 0.2	3.0 ± 0.1	2.0 ± 0.1	1.7 ± 0.1
100 µM 3HO	11.6 ± 1.4	1.3 ± 0.2	4.2 ± 0.5	3.0 ± 0.2	2.3 ± 0.2	3.0 ± 0.1	3.3 ± 0.1	1.5 ± 0.1	2.7 ± 0.2	2.1 ± 0.2	3.3 ± 0.1	2.0 ± 0.1	1.9 ± 0.1
100 µM 3HDec	8.0 ± 0.8	1.1 ± 0.1	3.0 ± 0.4	2.1 ± 0.2	2.0 ± 0.2	1.7 ± 0.1	2.2 ± 0.1	1.1 ± 0.1	2.5 ± 0.2	1.8 ± 0.1	2.2 ± 0.1	1.6 ± 0.1	1.4 ± 0.1
400 µM 3HDec	n.d.	n.d.	n.d.	2.6 ± 0.2	2.7 ± 0.1	2.8 ± 0.1	2.8 ± 0.1	1.3 ± 0.1	2.6 ± 0.2	2.2 ± 0.2	2.8 ± 0.1	2.0 ± 0.2	1.8 ± 0.1
**GPR84**													
25 µM C10	n.d.	n.d.	n.d.	3.6 ± 0.3	3.5 ± 0.6	2.1 ± 0.3	3.5 ± 0.1	1.6 ± 0.1	3.6 ± 0.3	2.8 ± 0.3	3.3 ± 0.1	3.7 ± 0.1	2.0 ± 0.1
100 µM C10	10.6 ± 1.7	1.7 ± 0.1	3.3 ± 0.3	4.7 ± 0.3	4.7 ± 0.3	3.7 ± 0.4	3.6 ± 0.1	1.5 ± 0.1	4.7 ± 0.3	3.4 ± 0.1	3.7 ± 0.1	4.0 ± 0.2	2.5 ± 0.1
25 µM 3HDec	n.d.	n.d.	n.d.	4.4 ± 0.3	4.5 ± 0.7	4.3 ± 0.2	4.1 ± 0.4	1.7 ±0.1	4.4 ± 0.3	4.3 ± 0.2	3.9 ± 0.2	4.0 ± 0.1	3.2 ± 0.2
100 µM 3HDec	9.6 ± 2.1	2.4 ± 0.5	2.9 ± 0.2	4.8 ± 0.5	4.8 ± 0.1	5.1 ± 0.1	4.5 ± 0.4	1.7 ± 0.1	4.8 ± 0.5	4.4 ± 0.4	4.2 ± 0.2	4.4 ± 0.3	3.4 ± 0.1

Supplementary Table S5

TPM values as downloaded from https://www.ebi.ac.uk/gxa/home [1]

organism part/tissue/cell type	Expression Atlas	HCAR3	GPR84
blood	E-MTAB-5214	42	17
neutrophil, mature	E-MTAB-3827	103	61
monocyte, CD14-positive, CD16-negative classical	E-MTAB-3827	82	30
neutrophil, segmented of bone marrow	E-MTAB-3827	58	15
macrophage, inflammatory	E-MTAB-3827	5	27
macrophage	E-MTAB-3827	3	3
metamyelocyte, neutrophilic	E-MTAB-3827	0.5	61
vermiform appendix	E-MTAB-2836	24	22
bone marrow	E-MTAB-2836	20	30

Experiments included: E-MTAB-2836 (Raw Data Provider: The Human Protein Atlas), E-MTAB-5214 (The Genotype-Tissue Expression (GTEx) pilot analysis) [2],61 E-MTAB-3827 (These studies make use of data generated by the Blueprint Consortium. A full list of the investigators who contributed to the generation of the data is available from www.blueprint-epigenome.eu. Funding for the project was provided by the European Union's Seventh Framework Programme (FP7/2007-2013) under grant agreement no 282510 – BLUEPRINT.),

Supplementary Results and Discussion

The human HCA_3 differs from gorilla HCA_3 only in three amino acids and a C terminus extended by 24 amino acids. The orangutan HCA_3 has only two differences compared to the gorilla HCA_3 (Tyr^{86}; Trp^{142}) (Figure S9D). We found that only for orangutan HCA_3 we do not observe similar differences between 3HO- and 3HDec-induced DMR responses in presence of dynasore compared to human HCA_3 (Figure S9). Thus, neither the extended C terminus nor the human HCA_3 specific amino acids Val^{156}, His^{253} and Ile^{317} are relevant for the observed reduction in 3HDec-induced DMR response in the presence of dynasore since gorilla HCA_3 DMR responses to different agonists are similarly affected by dynasore. This suggests a role of Tyr^{86} and/or Trp^{142} for the observed effect of dynasore. Asn^{86} of human HCA_2 in TM2/ECL1 has been shown to be crucial for nicotinic acid binding [3]. Tyr^{86} of HCA_3 might more likely play an indirect role for the interaction of HCA_3 with dyn-2 whereas Trp^{142}, located at the transition of ICL2 to TM4, could be relevant for a direct interaction. The finding that presence of dynasore causes a sustained signaling of the evolutionarily closest HCA_3-relatives HCA_1 (Arg^{130}) and HCA_2 (Arg^{142}) further supports this hypothesis (Figure S4). DMR analyses of the gorilla and orangutan HCA_3 with D-PLA, ILA, D-Phe and L-PLA revealed that activation profiles and dynasore sensitivity of the DMR response are conserved between human and gorilla HCA_3 (Figure S9). In contrast, orangutan HCA_3 is less potently activated by all agonists tested and presence of dynasore rather causes sustained or unchanged signaling than inhibition (Figure S9C).

In summary, our analyses showed that dynasore affected the DMR response of human and gorilla but not orang utan HCA_3 to D Phe and L-PLA in a similar manner like that to 3HDec, whereas the DMR response of D-PLA and ILA was similarly affected like that one to 3HO (Figure S9). Thus, the crucial involvement of dyn-2 in HCA_3 signaling and trafficking is a feature acquired during evolution before the split of the human/chimpanzee/gorilla lineage.

References

1. Petryszak R, Keays M, Tang YA, Fonseca NA, Barrera E, Burdett T, Fullgrabe A, Fuentes AM, Jupp S, Koskinen S, et al: **Expression Atlas update--an integrated database of gene and protein expression in humans, animals and plants.** *Nucleic Acids Res* 2016, **44:**D746-752.
2. Carithers LJ, Moore HM: **The Genotype-Tissue Expression (GTEx) Project.** *Biopreserv Biobank* 2015, **13:**307-308.
3. Tunaru S, Lattig J, Kero J, Krause G, Offermanns S: **Characterization of determinants of ligand binding to the nicotinic acid receptor GPR109A (HM74A/PUMA-G).** *Mol Pharmacol* 2005, **68:**1271-1280.
